# Optimizing Japanese dietary patterns to improve nutritional quality, reduce environmental impact under affordability constraints in younger and older adults from the nationwide MINNADE study

**DOI:** 10.1007/s00394-026-04129-x

**Published:** 2026-10-13

**Authors:** Minami Sugimoto, Hitomi Okubo, Yasunori Kikuchi, Sander Biesbroek, Elisabeth H. M. Temme, Ryoko Katagiri

**Affiliations:** 1 Department of Environmental and Occupational Health, School of Medicine, Toho University, 5-21-16 Omori-Nishi, Ota-Ku, Tokyo 143-8540 Japan; 2 Institute for Future Initiatives, University of Tokyo, 7-3-1 Hongo, Bunkyo-ku, Tokyo 113-0033 Japan; 3 Department of Nutritional Epidemiology and Behavioural Nutrition, Graduate School of Medicine, The University of Tokyo, 7-3-1 Hongo, Bunkyo-ku, Tokyo 113-0033 Japan; 4 Department of Chemical System Engineering, The University of Tokyo, 7-3-1 Hongo, Bunkyo-ku, Tokyo 113-8656 Japan; 5 Presidential Endowed Chair for “Platinum Society”, The University of Tokyo, 7-3-1 Hongo, Bunkyo-ku, Tokyo 113-8656 Japan; 6 Division of Human Nutrition and Health, Wageningen University & Research, 6700 AA Wageningen, The Netherlands; 7 Department for Healthy and Sustainable Nutrition, Centre for Prevention, Lifestyle and Health, National Institute for Public Health and the Environment (RIVM), 3721 MA Bilthoven, the Netherlands; 8 Faculty of Informatics, Graduate School of Informatics, Chiba University, 1-33, Yayoicho, Inage-ku, Chiba-shi, Chiba 263-8522 Japan

**Keywords:** Data envelopment analysis, Sustainable diet, Diet optimisation, Environmental impact, Dietary reference intakes for Japanese

## Abstract

**Purpose:**

To optimise diets that are more nutritious, have lower diet-related greenhouse gas emissions (GHGE) based on cradle-to-pre-retail impact, and remain affordable for Japanese adults of different age groups.

**Methods:**

Eight-day dietary records collected from younger (aged 18–49 years; 711 males and 734 females) and older (aged 50–74 years; 565 males and 605 females) Japanese adults were analysed. For each sex–age group, optimised diets were derived under three optimisation models using Data Envelopment Analysis: (i) MaxNRF: maximisation of diet quality score (Nutrient Rich Food Index [NRF] 9.3); (ii) MinGHGE: minimisation of GHGE; and (iii) OPT2G: combination of both objectives. All models included a monetary cost constraint (105% of observed). The proportions of participants having inadequate nutrient intakes, based on the Japanese Dietary Reference Intakes, were compared between observed diets and three optimised diets.

**Results:**

All optimised diets showed NRF9.3 scores 13.5–35.9% higher than those observed in all sex-age groups. GHGE decreased by 12.6–32.7% in the MinGHGE and OPT2G only. Differences between the optimised and observed diets in NRF9.3 score and GHGE were generally larger in younger than in older adults. All optimised diets showed lower or similar prevalences of inadequate nutrient intake compared with observed values. However, in younger males, optimised diets with MinGHGE had less favourable macronutrient distributions and higher inadequacies of vitamins A, C, and riboflavin than observed.

**Conclusion:**

Optimised diets showed better nutrient adequacy and lower GHGE across all sex-age groups, although specific nutrient shortfalls warrant attention when minimising GHGE in younger males.

**Supplementary Information:**

The online version contains supplementary material available at https://doi.org/10.1007/s00394-026-04129-x.

## Introduction

A suboptimal diet is the leading preventable risk factor for non-communicable diseases [1]. Healthy dietary patterns can reduce disease risk and mortality, and are therefore central to public health strategies [2]. Many countries have developed Food-Based Dietary Guidelines (FBDGs) that propose recommendations for foods, food groups, and dietary patterns to promote healthy eating at the population level [3]. Recently, an increasing number of countries have incorporated environmental sustainability into their FBDGs [4]. This reflects growing concerns about the substantial contribution of the food system to greenhouse gas emissions (GHGE), land use change, water use and other environmental burdens [4–6]. Thus, future FBDGs will be expected to incorporate perspectives on environmental sustainability in addition to health considerations, consistent with recommendations from international organisations, exemplified by the Food and Agriculture Organization of the United Nations and World Health Organization (WHO) [7]. For example, FBDGs in Denmark [8] and Brazil [9] incorporate sustainability considerations.

In Japan, diet-related health challenges are prominent and show clear sex- and age-specific patterns. Younger adults often consume excessive amounts of fats and sugars while consuming insufficient amounts of fruits and vegetables [10]. A substantial proportion of young female adults are underweight (BMI < 18.5 kg/m^2^) and have inadequate intake of calcium and iron [10, 11], while the prevalence of obesity has been rising in male adults [10]. Across both sexes, excessive energy intake from total and saturated fat and high sodium intake are common [10, 11]. Although nutrient-based guidance is available through the Dietary Reference Intakes (DRIs) for Japanese [12], Japan currently lacks a comprehensive dietary guideline at the food level. In addition, the existing dish-based guideline, the Japanese Food Guide Spinning Top, has not been substantially updated for over 20 years and does not incorporate environmental considerations [13]. The development of updated FBDGs that integrate food- or dish-level guidance with both health and environmental perspectives is therefore critical to supporting dietary improvement in Japan.

Toward the development of FBDGs, mathematical optimisation methods have been increasingly applied to dietary intake data to identify patterns that meet nutrient requirements, reduce environmental impact, or improve affordability [14–19]. Among these, data envelopment analysis (DEA)-based models, such as the SHARP (Sustainable, Healthy, Affordable, Reliable, and Preferable diets) model, construct optimised diets as linear combinations of observed diets [20–24]. This model has the advantage of producing feasible and culturally acceptable dietary patterns [20]. However, previous applications of the SHARP model have lacked age-specific analyses [20–24] and have insufficiently assessed nutrient adequacy at the individual nutrient level [20–23]. In addition, previous applications in the Japanese population have been limited by small sample sizes [23]. Consequently, the feasibility and nutritional quality of optimised diets across Japanese adults of different ages remain unclear.

Here, we aimed to identify dietary patterns for Japanese adults that are more nutritious, environmentally sustainable, and affordable across different age groups, and to evaluate the nutrient adequacy of these optimised diets using DRIs for Japanese. To produce feasible and culturally acceptable dietary patterns, we leveraged a DEA-based model. By addressing sex- and age-specific dietary challenges and integrating environmental and cost considerations, this study aimed to produce findings to aid the development of comprehensive, sustainable FBDGs in Japan, and potentially across the broader Asian region.

## Methods

### Dietary data

#### Study design and participants

Dietary data are from the MINNADE (MINistry of Health, Labour and Welfare-sponsored NAtionwide study on Dietary intake Evaluation) survey, which involved three rounds of one-year data collection (first round: November 2016–September 2017; second: October 2017-September 2018; and third: November 2019-August 2020). Details of the survey have been reported previously [11, 25]. Participants were from 32 of Japan’s 47 prefectures, with suitable geographical diversity and representing 85% of the total population of Japan.

Research dietitians in the respective prefectures (n = 453) conducted participant recruitment and data collection. During the first survey wave, 256 participants (128 males and 128 females) were selected within each of nine age categories (1–6, 7–13, 14–19, 20–29, 30–39, 40–49, 50–59, 60–69, and 70–79 years), resulting in 2304 individuals overall. Considering attrition from this initial wave, the second round recruited 110–119 individuals per sex–age group, yielding a total of 2051 participants. In the third wave, an additional 438 children of ages 1–6 years were included for further data collection, but these data were not used in the current analysis.

Eligible participants at recruitment were community-dwelling individuals able to complete dietary records (DR) independently or, for children, with a guardian’s assistance. We excluded dietitians and people cohabitating with them, those working with research dietitians, those who had undergone dietary counselling, patients on insulin therapy or dialysis, pregnant and lactating women, and infants who were exclusively fed human milk. Participants were recruited non-randomly, with only one person per household included.

For the present analysis, participants aged ≥18 and <75 years were included; thus, participants aged 18 and 19 years from the 14 to 19-year recruitment category were also eligible. Excluding dropouts, 2894 adults aged 18 years and older provided ≥1-day DR data. Among these, we excluded 193 adults aged >74 years, 8 adults with fewer than 8 days of DR, 61 with consecutive DR data, 5 who completed the DR outside the designated months (e.g., October to December for autumn), and 12 pregnant and lactating women. Consequently, the current analysis included 711 males and 734 females aged 18–49 years (younger group) and 565 males and 605 females aged 50–74 years (older group).

The MINNADE study was conducted in accordance with the Declaration of Helsinki and obtained approval from the Ethics Committee of the University of Tokyo Faculty of Medicine (protocol code: 11,397). All participants provided written informed consent. The dataset of the MINNADE study is currently administered by the Consumer Affairs Agency, Government of Japan. Permission to use the anonymised dataset of the MINNADE study was obtained from the Consumer Affairs Agency in December 2024. The study protocol involving the secondary use of the MINNADE study dataset obtained approval from the Ethics Review Committee of the Graduate School of Engineering, the Graduate School of Informatics, the Design Research Institute, and the Frontier Medical Engineering Center, Chiba University (approval no. R6-26 on February 18, 2025).

#### Dietary assessment

Dietary intake was assessed via an 8-day weighed DR, collected over two non-consecutive days in each of spring, summer, autumn, and winter i.e., the four seasons (8 days in total). Details of the DR method were reported previously [11, 25]. In brief, in each season, half of the participants recorded intake on two weekdays, while the second half recorded their intake on one weekday and one weekend day, to approximate habitual intake with a weekday-to-weekend ratio of about 3:1. Research dietitians provided verbal and written instructions, and participants were asked to weigh every food and beverage consumed using a digital kitchen scale (KS-812WT, Tanita, Tokyo, Japan) and record them.

Research dietitians collected the recording sheets within a few days after each recording day and checked them for completeness, adding details when necessary. Each food item was assigned a code based on the Standard Tables of Food Composition in Japan (hereafter, “Japanese Food Composition Tables”) [26]. All food codes and weights were later verified by other research dietitians at the study’s central office. Daily energy and nutrient intakes from foods and beverages, excluding dietary supplements, were then calculated using the Japanese Food Composition Tables, 2015 edition [26]. The 2015 edition was used to maintain methodological consistency with the DRIs used for assessing nutrient adequacy, given the substantial methodological revisions introduced in the 2020 edition, particularly for energy and dietary fibre.

### Variables for analysis

#### Nutrient and food intake

Food items included in the Japanese Food Composition Tables were classified into 21 food groups defined in Supplemental Table 1.

**Table 1:** Comparison of basic characteristics between male participants with efficient diets and those with inefficient diets*

	Younger males (aged 18–49 years; n=711)					Older males (aged 50–74 years; n=565)				
	Efficient diets (n=131)		Inefficient diets (n=580)		*P*-value†	Efficient diets (n=131)		Inefficient diets (n=434)		*P*-value†
	Mean or N	SD or (%)	Mean or N	SD or (%)		Mean or N	SD or (%)	Mean or N	SD or (%)	
Age (years)	33.5	8.7	33.4	8.9	0.93	64.1	7.3	61.7	7.2	0.001
Body height (cm)	170.9	6.0	171.3	6.0	0.50	166.7	5.3	168.4	5.7	0.004
Body weight (kg)	69.0	12.6	68.9	12.6	1.0	66.7	9.5	67.7	8.8	0.26
BMI (kg/m^2^)	23.5	3.6	23.5	3.9	0.83	24.0	3.1	23.9	2.8	0.75
*Weight status*										
Underweight (BMI: <18.5 kg/m^2^)	5	(4)	30	(5)	0.73	0	(0)	7	(2)	0.28
Normal (BMI: 18.5–24.9 kg/m^2^)	86	(66)	387	(67)		87	(66)	296	(68)	
Overweight or obesity (≥ 25 kg/m^2^)	40	(31)	163	(28)		44	(34)	131	(30)	
Residential area, N (%)										
Hokkaido and Tohoku	15	(11)	64	(11)	0.30	9	(7)	55	(13)	0.40
Kanto	50	(38)	177	(31)		45	(34)	133	(31)	
Hokuriku and Tokai	19	(15)	121	(21)		25	(19)	90	(21)	
Kinki, Chugoku and Shikoku	33	(25)	140	(24)		35	(27)	108	(25)	
Kyushu and Okinawa	14	(11)	78	(13)		17	(13)	48	(11)	
Annual household income, N (%)										
<5 million Japanese yen	53	(40)	245	(42)	0.33	67	(51)	191	(44)	0.46
≥5 to <8 million Japanese yen	42	(32)	190	(33)		35	(27)	133	(31)	
≥8 million Japanese yen	36	(27)	134	(23)		29	(22)	108	(25)	
Missing	0	(0)	11	(2)		0	(0)	2	(0)	
Educational level, N (%)										
Junior high or high school	30	(23)	178	(31)	0.44	51	(39)	201	(46)	0.49
Junior college or technical school	38	(29)	153	(26)		16	(12)	52	(12)	
University or higher	62	(47)	242	(42)		63	(48)	178	(41)	
Other	0	(0)	2	(0)		1	(1)	3	(1)	
Missing	1	(1)	5	(1)			(0)		(0)	
Employment status, N (%)										
Unemployed	1	(1)	5	(1)	0.58	51	(39)	96	(22)	0.0005
Students	8	(6)	48	(8)		0	(0)	0	(0)	
Part-time job	5	(4)	10	(2)		18	(14)	67	(15)	
Full-time job	116	(89)	513	(88)		62	(47)	271	(62)	
Missing	1	(1)	4	(1)			(0)		(0)	
*Smoking status, N (%)*										
Current smoker	19	(15)	177	(31)	0.0019	15	(11)	111	(26)	0.0008
Past smoker	26	(20)	113	(19)		69	(53)	218	(50)	
Non-smoker	85	(65)	286	(49)		47	(36)	105	(24)	
Missing	1	(1)	4	(1)		0	(0)	0	(0)	
Energy intake (kcal/day)	2100	561	2235	516	0.008	2202	444	2310	432	0.01
NRF9.3 score	648.6	133.4	573.2	109.3	<0.0001	728.2	77.8	678.1	82.2	<0.0001
Greenhouse gas emissions (kgCO_2_-eq/day)‡	3.60	0.71	3.64	0.79	0.62	3.69	0.59	3.81	0.63	0.29
Acidification (gSO_2_-eq/day) ‡	3.1	1.2	2.9	1.0	0.05	3.4	1.0	3.3	1.1	0.53
Eutrophication (gPO_4_^3−^-eq/day) ‡	97.5	16.1	100.1	22.1	<.0001	92.2	13.0	92.5	15.2	0.021
Land use change (m^2^/day) ‡	0.049	0.008	0.051	0.01	<.0001	0.049	0.006	0.050	0.01	0.007
Water resources consumption (L/day) ‡	2200	705	2172	599	0.024	2303	711	2335	762	0.31
Monetary diet cost (Japanese yen/day) ‡	1189	204	1242	247	0.003	1357	216	1438	273	0.0005

BMI, body mass index; NRF, Nutrient Rich Food index

^*^Efficient diets were identified separately for 711 younger males aged 18–49 years and 565 older males aged 50–74 years using data envelopment analysis of 8-day dietary record data

^†^*P* values were obtained using Student’s t-test for continuous variables and chi-squared test for categorical variables. *P*<0.05 was considered statistically significant

^‡^Values per 2500 kcal

Daily nutrient and food intakes were calculated as the mean of the 8 recording days and then standardised to the same energy level (i.e., 2500 kcal for males and 2000 kcal for females) for application of the DEA-diet model, consistent with previous studies [21, 23].

#### Nutrient intake quality

Nutrient intake quality of the overall diet was assessed with an adapted version of the Nutrient Rich Food Index (NRF) 9.3 [27–29], which is calculated as the sum of the percentages of reference daily values (RDVs) of nine qualifying nutrients minus the sum of the percentages of RDVs of three disqualifying nutrients. To quantify the nutrient density based on the recommendation for Japanese, RDVs were determined (for sex and age categories) based on the DRIs for Japanese, 2025 [12], namely the Recommended Dietary Allowance (RDA) for protein, vitamins A and C, calcium, iron, and magnesium, the Adequate Intake (AI) for vitamin D, and the Tentative Dietary Goal for Preventing Lifestyle-related Diseases (DG) for dietary fibre, potassium, saturated fats and sodium. Regarding added sugars, WHO’s conditional recommendation (i.e., upper limit of 5% of energy) [30] was adopted because a recommended value for added sugar does not exist in Japan.

Reference values in Dietary Reference Intakes for Japanese are defined as follows: RDA is defined as “an estimate of the daily average dietary intake that satisfies the needs of most of the individuals belonging to a population (97–98%), on the basis of the distribution of the measured requirements of a study population.”; AI, “a recommended average daily-nutrient-intake level based on observed or experimentally determined approximations or estimates of nutrient intake by a group (or groups) of apparently healthy people that are assumed to be adequate”; and DG, “the average daily-nutrient-intake level (or ranges) that Japanese should currently aim to consume primarily to prevent chronic diseases [12].” The RDVs used are listed in Supplementary Table S2.

#### Diet-related environmental impact from food production

**Fig. 1:**
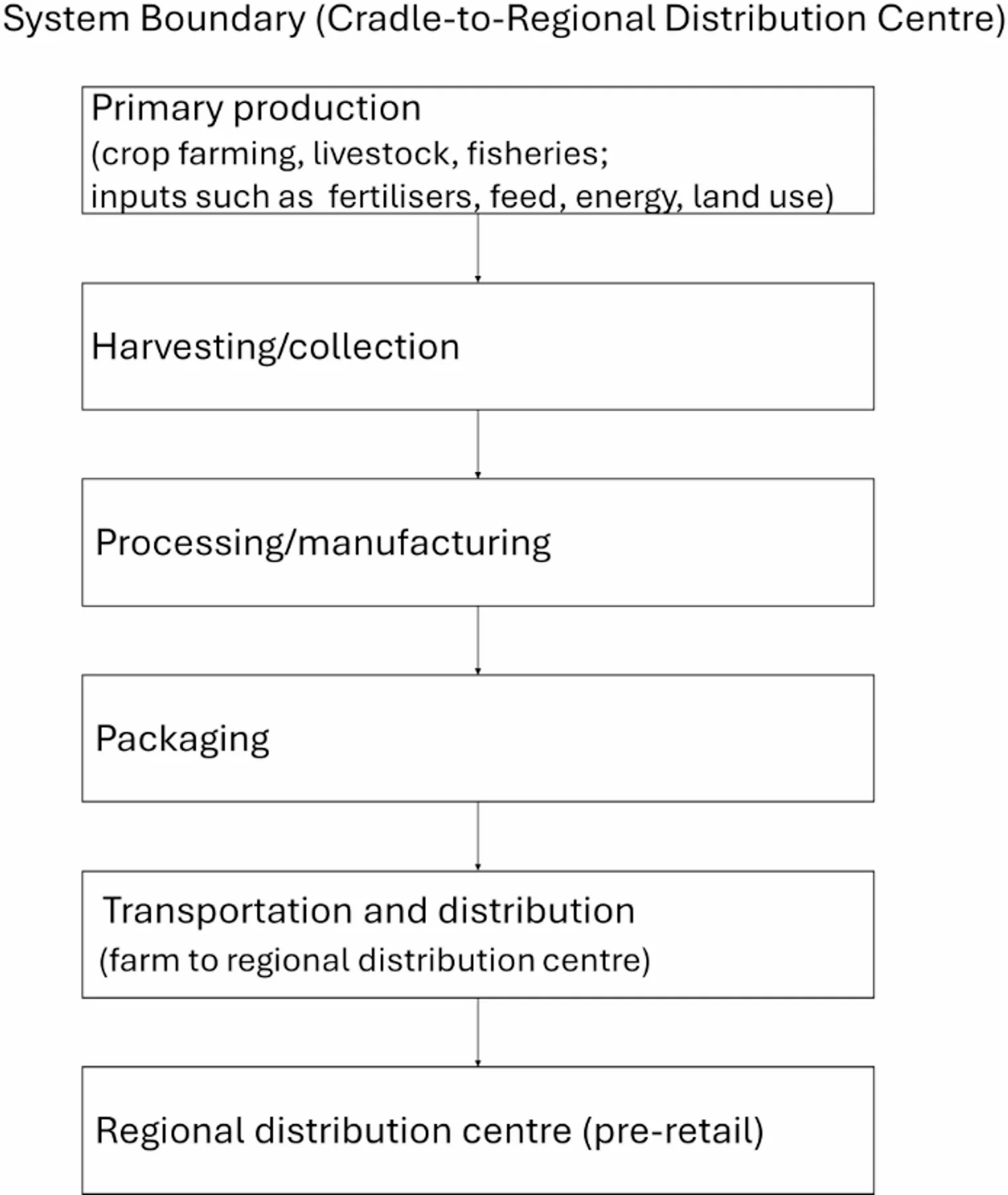
System boundary of diet-related environmental impact

Diet-related environmental impact was estimated using IDEA version 3.4.1, developed by The Research Institute of Science for Safety and Sustainability, Japan (https://riss.aist.go.jp/en-idealab/) [31]. The system boundary of estimation was cradle-to-(regional) distribution centre (pre-retail) (Fig. 1). IDEA was developed using a hybrid life cycle assessment method and contains life cycle inventory data for products and services in Japan and raw process inventory data. The results of life cycle inventory analysis can be converted into life cycle impact categories by applying a Japanese life cycle impact assessment method, the Life Cycle Impact Assessment Method based on the Endpoint modelling version 2 (LIME2) [32]. The database’s system boundaries span from farms and production to regional distribution centres. In IDEA, domestic production was assumed for all commodities. Values of environmental indicators in IDEA version 3.4.1 were linked to food codes in Standard Tables of Food Composition in Japan [26] via a correspondence table [33]. Because the number of food items in IDEA (approximately 350) was far fewer than in STFC Japan (> 2000), a closely related food item was assigned for items without an exact match in IDEA when available (e.g., the GHGE value for “onion” was used for “red onion”). Otherwise, an aggregated category was used (e.g., the GHGE value for “other root vegetables” was used for “ginger”). The following indicators were selected based on relevance to the food sector, availability of data for food-related environmental impacts, and reliability of the data [2]: GHGE (kgCO_2_-eq), eutrophication (gPO_4_^3−^-eq), acidification (gSO_2_-eq), land use change (land transformation; assuming transformation from forest) (m^2^), and water resources consumption (including withdrawal and consumptive and non-consumptive water use; blue and green water; regional factors not considered) (L). All indicators were expressed using a functional unit of daily energy intake per person, standardised to 2500 kcal for males and 2000 kcal for females.

#### Monetary cost of diet

The monetary cost of the diet was estimated to indicate dietary affordability. Dietary data were linked to retail food prices, obtained mainly from the National Retail Price Survey 2013, which measures average prices per 100 g for major food items, and supplemented with price information from a major supermarket. The National Retail Price Survey is an annual national survey performed by the Statistics Bureau, Ministry of Internal Affairs and Communications. Details of the calculation method have been provided elsewhere [23, 34]. Price data from The National Retail Price Survey were associated with Japanese Food Composition Tables for food items appearing in the 2013 National Health and Nutrition Survey [34], and then extended to all food items in another study [23]. The monetary cost of the diet [JPY: Japanese yen] was also calculated per 2500 kcal for males and 2000 kcal for females.

#### Other variables

Participants’ body weight and height were measured in light indoor clothing and barefoot by family or research dietitians by standardised procedures to the nearest 0.1 kg and 0.1 cm, respectively, at the beginning of each survey round. For those whose weight or height (n=17) could not be measured or whose measured values appeared incorrect, self-reported information was used. Body Mass Index (BMI) was calculated as body weight (kg) divided by the body height squared (m^2^). Other data on the basic characteristics were collected by questionnaire. Age (years) at the study start was calculated from the date of birth. Weight status was categorised as underweight (BMI<18.5 kg/m^2^), normal (18.5–24.9 kg/m^2^), and overweight or obesity (≥ 25 kg/m^2^).

Residence area was grouped into 1 of 5 regions (Hokkaido and Tohoku; Kanto; Hokuriku and Tokai; Kinki, Chugoku and Shikoku; or Kyushu and Okinawa). Annual household income was categorised as <5 million Japanese yen, ≥5 to <8 million Japanese yen, ≥8 million Japanese yen, or missing. Educational attainment was classified as junior high or high school, junior college or technical school, university or higher, other, or missing. Employment status was categorised as student, unemployed, part-time, full-time, or missing. Smoking habit of adults was categorised as current smoker, past smoker, non-smoker, or missing.

### Calculation of optimised diets by the data envelopment analysis (DEA) diet model

**Fig. 2:**
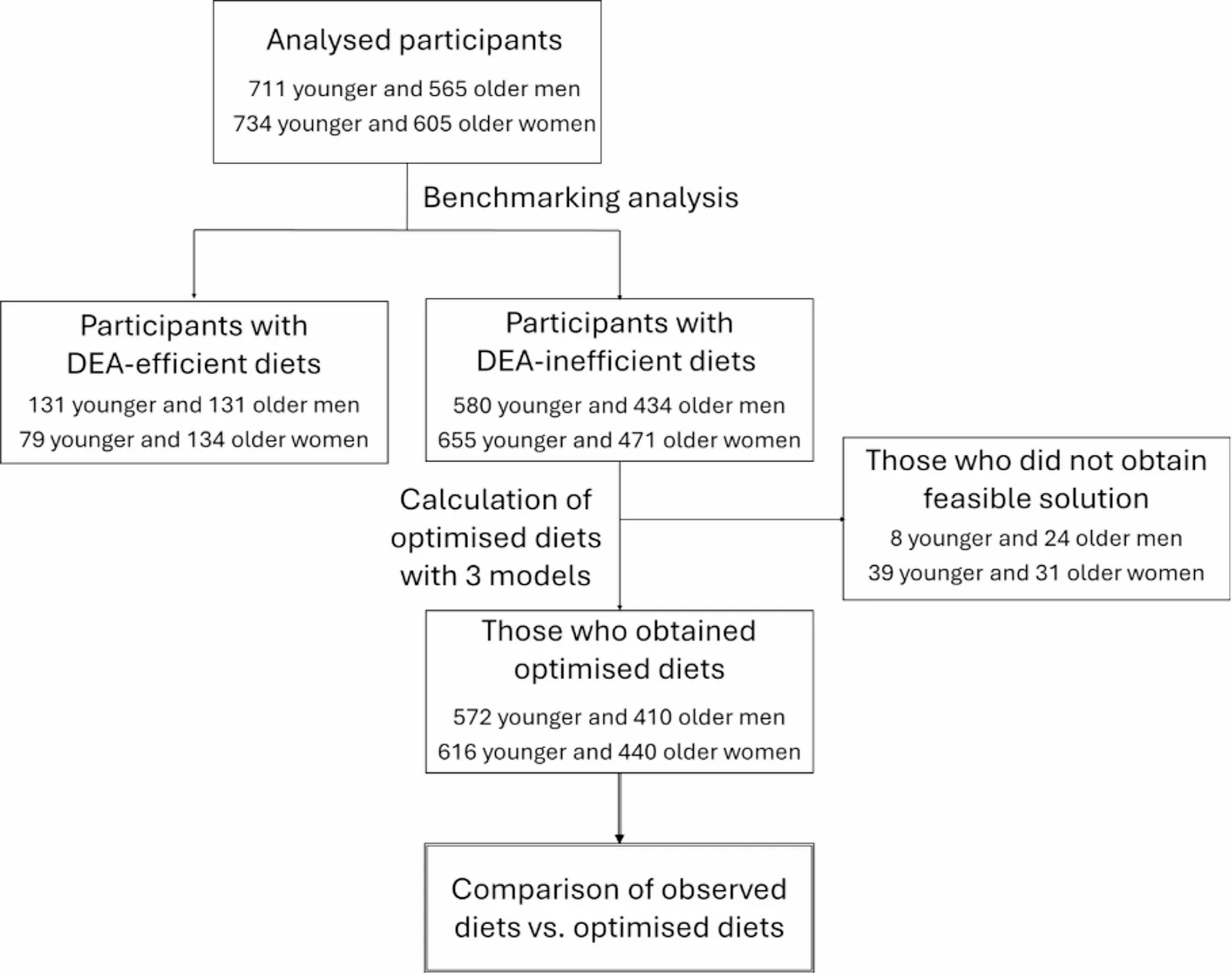
Flow chart of analysed participants. DEA, Data Envelopment Analysis. Participants with efficient diets were identified by benchmarking analysis. The remaining participants were categorised as inefficient diets. For those with inefficient diets, optimised diets were calculated using three models. Some participants who did not obtain a feasible solution across all three models were excluded from the analysis comparing observed and optimised diets. No participant failed to obtain a feasible solution in one or two models only. Younger males (n=711) and females (n=734) aged 18–49 years and older males (n=565) and females (n=605) aged 50–74 years were analysed

**Fig. 3:**
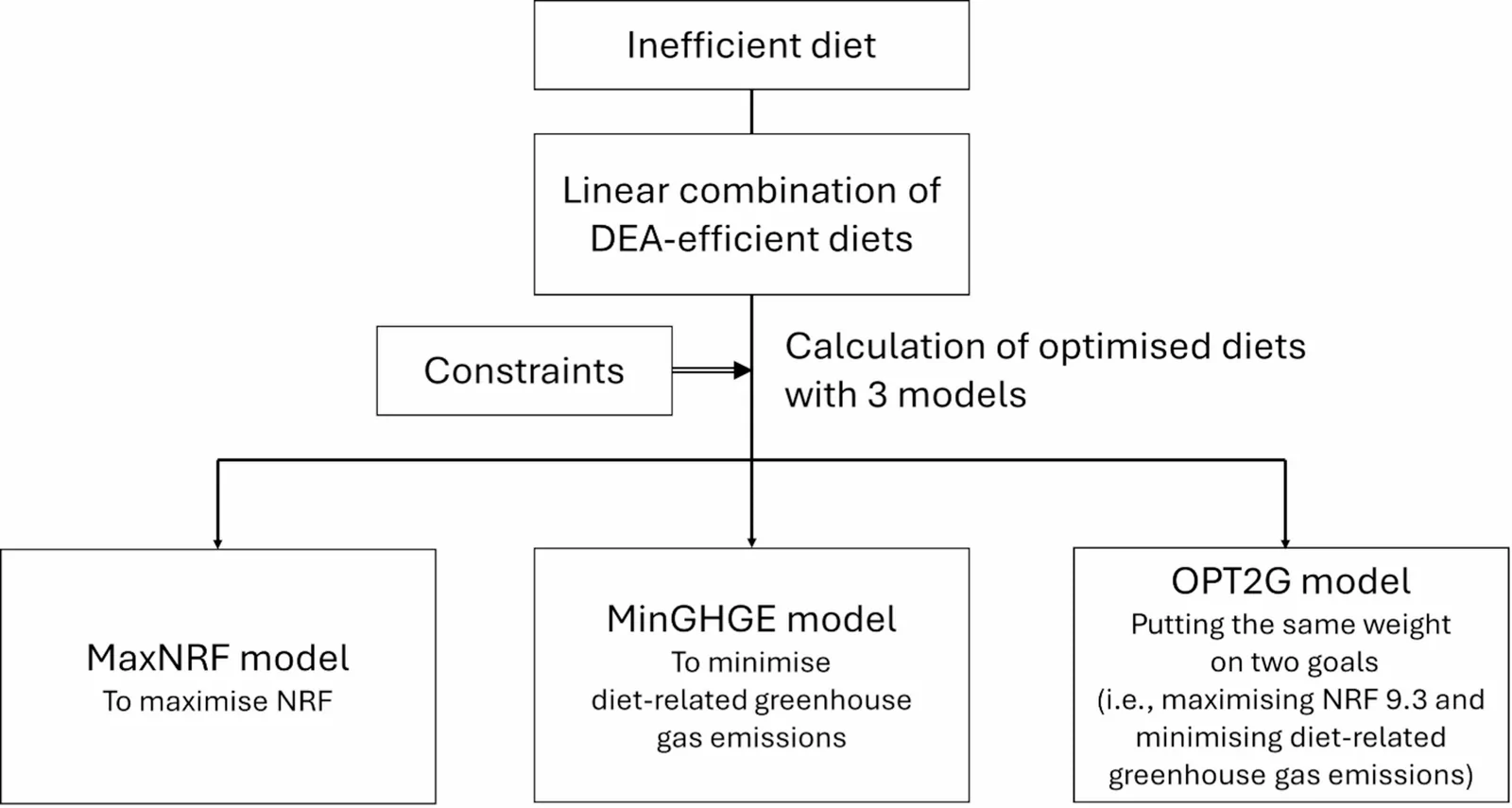
Overview of the calculation of the optimised diets using the Data Envelopment Analysis diet model. An optimised diet was calculated as a linear combination of observed diets for each individual identified as having an “inefficient diet.” MaxNRF model: optimised diets targeted to maximise the Nutrient Rich Food index 9.3 score. MinGHGE model: optimised diets targeted to minimise diet-related greenhouse gas emissions. OPT2G model: Optimised diets targeted to achive two goals (i.e., maximise the Nutrient Rich Food index 9.3 score and minimise diet-related greenhouse gas emissions) with equal weight

Optimised diets were calculated using the DEA diet model following a two-step procedure (Figs. 2 and 3) per person: (1) identification of “efficient diets” by benchmarking analysis, and (2) derivation of alternative optimised diets for individuals with “inefficient diets”. Here, an efficient diet refers to a diet with a relatively favourable overall combination of intakes of food groups or nutrients recommended for consumption and those recommended to be limited, compared with the diets of other participants. Observed and optimised diets were then compared among participants with inefficient diets, while those who could not obtain a feasible solution were excluded from the comparison (Fig. 2). Analyses were conducted with stratification by sex and age group (younger [aged 18–49 years] and older [aged 50–74 years] groups).

The theoretical background of this approach is reported elsewhere [20, 23]. The complete formulas for the models are provided in Appendices A–C. All analyses were conducted using FICO® Xpress-Workbench version 3.16.

#### Identification of the efficient diets

We first conducted a benchmarking analysis to identify “efficient diets” that achieved a favourable balance between “dietary components to increase” and “dietary components to decrease”. Dietary components and their classification as components to increase or decrease were determined according to the food-group components and scoring direction of the Diet Quality Score for Japanese (DQSJ) [35]. Components to increase included fruits, vegetables, legumes, nuts and seeds, dairy products, oily and lean fish (excluding dried, canned and processed fish), and whole grains. Components to decrease included red and processed meat products, sodium, sugar-sweetened beverages, and alcoholic beverages. Alcoholic beverages were further included to prevent the misclassification of high-alcohol diets as efficient, although alcoholic beverages were not included in the DQSJ.

An input–output DEA model based on the Banker, Charnes, and Cooper framework was applied [36]. All dietary components were standardised to a 0–1 scale to minimise the influence of variables with large absolute intakes. Zero intakes were replaced with half of the lowest non-zero intake within each sex and age group, solely for the benchmarking analysis to avoid zero denominators in intake ratios. An efficiency score (θ) was calculated for each participant. Diets with θ = 1 were classified as efficient, while all others were labelled inefficient (Fig. 2). To summarise the dietary intake profiles of efficient and inefficient diets, a descriptive comparison is provided in Supplementary Tables 4 and 5.

#### Calculation of optimised diets

For each participant with an inefficient diet, optimised diets were generated as a linear combination of efficient diets (Fig. 3). By varying the proportional contribution of each efficient diet (${\lambda}_{k}$), alternative diets optimising predefined objectives were obtained. The proportion of efficient diets (${\lambda}_{k}$) in the combinations was determined by calculating the linear programming for optimisation models:

1. MaxNRF model: maximisation of the NRF9.3 score
2. MinGHGE model: minimisation of diet-related GHGE
3. OPT2G model: simultaneous consideration of nutritional quality (NRF9.3) and diet-related GHGE

For OPT2G, both NRF9.3 and diet-related GHGE were standardised by dividing by their respective population ranges (maximum – minimum). The optimisation objective was to maximise the difference between the standardised NRF9.3 score and the standardised diet-related GHGE.

Several constraints were imposed to ensure nutritional plausibility and real-world feasibility:

1. Intake directionality: dietary components to increase (or decrease) in the optimised diet had to be higher (or lower) than in the observed diet.
2. Energy balance: total energy intake matched each participant’s observed intake (i.e., 2500 kcal for males and 2000 kcal for females).
3. Vitamin A: intake was restricted to below the sex-specific 95th percentile of observed values to avoid unrealistic values.
4. Diet cost: the monetary cost of optimised diets was required to be ≤105% of the observed cost for each individual. A 5% margin was allowed because stricter cost constraints (i.e., the same or less than observed) resulted in no feasible solutions for 196 participants.

For each participant with an inefficient diet, food and nutrient intakes and diet-related environmental indicators of optimised diets were calculated as linear combinations of those variables among efficient diets. The NRF9.3 score of optimised diets was calculated using nutrient intakes and sex-age-specific RDVs for each participant. We did not impose additional acceptability constraints, such as limits on deviations from the observed diet. Because the optimised diets were derived as linear combinations of existing diets, they were expected to remain within dietary patterns observed in the study population to some extent.

### Nutrition intake inadequacy in observed and optimised diet

Nutrient intakes (per 2500 kcal or 2000 kcal) were compared against age- and sex-specific reference values from the DRIs for Japanese [12], as described previously [11, 37–39]. Briefly, several types of reference values are defined for their respective purposes in the Japanese DRIs. In addition to RDA and DG described above, the Estimated Average Requirement (EAR) is defined as “the estimated intake amount that meets the requirements of 50% of the individuals belonging to an age or sex group [12].” Proportions of inadequate nutrient intake were assessed as the percentage of participants who did not meet the DRIs using the cut-point method [40]. For the 13 nutrients with the EAR, intake levels below the EAR were considered inadequate: protein, vitamin A (retinol equivalents), thiamine, riboflavin, niacin (niacin equivalent), vitamin B_6_, folate, vitamin C, calcium, magnesium, iron, zinc, and copper [12]. For 7 nutrients with the DG, we calculated the percentages of participants consuming nutrient intakes outside (below or above) the range of the DG for the following 7 nutrients: protein (% energy), total fat (% energy), saturated fat (% energy), carbohydrates (% energy), dietary fibre, sodium, and potassium. For added sugar, participants with intakes of 5% or more of energy were deemed inadequate [30]. The reference values of EAR and DG are given in Supplementary Tables S3.

### Statistical analysis

Statistical analyses were conducted using SAS statistical software version 9.4 (SAS Institute Inc., Cary, NC, USA). All reported P-values were two-tailed, with a *P*-value < 0.05 considered statistically significant.

Descriptive statistics for participant characteristics, NRF 9.3 score, diet-related environmental indicators, monetary cost and intakes of food and nutrients in observed diets are presented as the mean (SD) for continuous variables and numbers (%) of participants for categorical variables. Comparisons between participants with efficient and inefficient diets were performed using the t-test for continuous variables and χ^2^ test for categorical variables. Similarly, characteristics of those who did and those who did not obtain feasible solutions were compared.

Among participants with an inefficient diet, the observed diet was compared with three types of optimised diets (MaxNRF, MinGHGE, OPT2G) using repeated-measures ANOVA (PROC GLM), followed by Bonferroni correction for multiple comparisons. Those with an inefficient diet who could not obtain a feasible solution were excluded from the comparison of optimised and observed diets. For each participant with an inefficient diet, the proportion of change in NRF 9.3 score, diet-related environmental indicators, and monetary cost was calculated as: Proportion of change (%)= ([values in optimised diets]—[values in observed diets])/[values in observed diets] × 100. For food group consumption, absolute differences in weight (g) were calculated for each participant, with percentage differences derived from mean intakes because some participants had zero intake in the observed diet.

The proportion of participants with inadequate intake under each optimised diet was compared with the proportion with inadequate intake in the observed diet and in another optimised diet using pairwise McNemar’s tests. Statistical significance was considered as *P* < 0.0083 (= 0.05/6, Bonferroni correction for six comparisons).

## Results

### Identification of efficient diets

Using the DEA diet model, 131 younger (18%) and 131 older (23%) males and 79 younger (11%) and 134 older (22%) females were identified as having efficient diets (Fig. 2). In older males and females, those with efficient diets were older than those with inefficient diets (e.g., [mean±SD] 64.1±7.3 vs 61.7±7.2 years in older males; Tables 1 and 2). Overall, participants with efficient diets were not significantly different from those having inefficient diets in demographic variables, except for body height (older males), smoking status (younger and older males), employment status (older males), and annual household income (older females). Dietary comparisons indicated that participants with efficient diets generally had lower energy intake before standardisation and higher NRF 9.3 scores than those with inefficient diets (e.g., 648.6±113.4 vs 573.2±109.3 in younger males). Specifically, those with efficient diets were characterised by higher intake of dietary components to increase, namely protein, total fibre, and several vitamins and minerals, and lower intake of components to decrease across all sex–age groups (Supplemental Tables S4 and S5). There was no difference in total and saturated fat, carbohydrate, or added sugar intake between those with efficient versus inefficient diets for all sex-age groups. There was no difference in diet-related GHGE in males (e.g., [mean±SD] 3.60±0.71 vs 3.64±0.79 kg CO_2_-eq in efficient and inefficient diets among younger males), while those with efficient diets had lower GHGE than those with inefficient diets among females (e.g., 2.86±0.50 vs 2.98±0.72 kg CO_2_-eq among younger females).

**Table 2:** Comparison of basic characteristics between female participants with efficient diets and those with inefficient diets*

	Younger females (aged 18–49 years; n=734)					Older females (aged 50–74 years; n=605)				
	Efficient diets (n=79)		Inefficient diets (n=655)		*P*-value†	Efficient diets (n=134)		Inefficient diets (n=471)		*P*-value†
	Mean or N	SD or (%)	Mean or N	SD or (%)		Mean or N	SD or (%)	Mean or N	SD or (%)	
Age (years)	34.5	9.6	33.2	9.4	0.22	64.2	6.9	61.5	7.4	0.0002
Body height (cm)	158.2	5.5	158.2	5.4	0.98	153.9	6.2	154.9	5.7	0.08
Body weight (kg)	54.1	9.8	54.4	9.2	0.79	53.8	8.9	55.3	8.9	0.07
BMI (kg/m^2^)	21.6	3.5	21.8	3.5	0.70	22.7	3.5	23.1	3.5	0.30
*Weight status*										
Underweight (BMI: <18.5 kg/m^2^)	11	(14)	76	(12)	0.59	9	(7)	27	(6)	0.19
Normal (BMI: 18.5–24.9 kg/m^2^)	55	(70)	491	(75)		102	(76)	328	(70)	
Overweight or obesity (≥ 25 kg/m^2^)	13	(16)	88	(13)		23	(17)	116	(25)	
*Residential area, N (%)*										
Hokkaido and Tohoku	7	(9)	78	(12)	0.33	14	(10)	50	(11)	0.048
Kanto	31	(39)	198	(30)		32	(24)	156	(33)	
Hokuriku and Tokai	12	(15)	133	(20)		27	(20)	88	(19)	
Kinki, Chugoku and Shikoku	17	(22)	170	(26)		36	(27)	130	(28)	
Kyushu and Okinawa	12	(15)	76	(12)		25	(19)	47	(10)	
*Annual household income, N (%)*										
<5 million Japanese yen	38	(48)	286	(44)	0.89	76	(57)	242	(51)	0.041
≥5 to <8 million Japanese yen	20	(25)	180	(27)		29	(22)	120	(25)	
≥8 million Japanese yen	19	(24)	174	(27)		26	(19)	108	(23)	
Missing	2	(3)	15	(2)		3	(2)	1	(0)	
*Educational level, N (%)*										
Junior high or high school	20	(25)	204	(31)	0.46	64	(48)	225	(48)	0.43
Junior college or technical school	32	(41)	273	(42)		55	(41)	188	(40)	
University or higher	27	(34)	175	(27)		12	(9)	53	(11)	
Other	0	(0)	3	(0)		3	(2)	3	(1)	
Missing	0	(0)	0	(0)		0	(0)	2	(0)	
*Employment status, N (%)*										
Unemployed	3	(4)	27	(4)	0.82	52	(39)	137	(29)	0.10
Students	10	(13)	64	(10)		0	(0)	0	(0)	
Part-time job	9	(11)	65	(10)		28	(21)	114	(24)	
Full-time job	57	(72)	499	(76)		54	(40)	220	(47)	
Missing	0	(0)	0	(0)		0	(0)	0	(0)	
*Smoking status, N (%)*										
Current smoker	3	(4)	77	(12)	0.08	8	(6)	25	(5)	0.82
Past smoker	7	(9)	70	(11)		13	(10)	54	(11)	
Non-smoker	69	(87)	508	(78)		113	(84)	392	(83)	
Energy intake (kcal)	1731	376	1765	339	0.41	1845	372	1912	297	0.031
NRF9.3 score	650	128	587	109	<0.0001	733	78	680	82	<0.0001
Greenhouse gas emissions (kgCO_2_-eq/day) ‡	2.86	0.50	2.98	0.72	<.0001	3.10	0.51	3.25	0.62	0.002
Acidification (gSO_2_-eq/day) ‡	2.29	0.74	2.37	0.83	0.14	2.59	0.71	2.68	0.78	0.21
Eutrophication (gPO_4_^3−^-eq/day) ‡	79.6	10.5	83.2	19.6	<.0001	77.9	10.4	79.0	11.5	0.15
Land use change (m^2^/day) ‡	0.041	0.005	0.043	0.008	<.0001	0.041	0.005	0.043	0.006	0.005
Water resources consumption (L/day) ‡	1686	508	1771	650	0.002	1925	530	2054	645	0.003
Monetary diet cost (Japanese yen/day) ‡	1003	164	1069	255	<.0001	1148	195	1228	221	0.063

BMI, body mass index; NRF, Nutrient Rich Food index

^*^Efficient diets were identified separately for 734 younger females aged 18–49 years and 605 older females aged 50–74 years using data envelopment analysis of 8-day dietary record data

^†^*P* values were obtained using Student’s t-test for continuous variables and chi-squared test for categorical variables. *P*<0.05 was considered statistically significant

^‡^Values per 2000 kcal

Across sex-age groups, those who did not obtain feasible solutions had lower monetary diet cost than those who did. There were no differences in BMI, educational level, employment status, and NRF 9.3 score between them in all sex-age groups (Supplemental Tables S6 and S7).

### Food composition of optimised diets

Among participants with inefficient diets, 102 adults who could not obtain feasible solutions were excluded from comparison of observed and optimised diets (8 younger and 24 older males and 39 younger and 31 older females), leaving 572 younger and 410 older males and 616 younger and 440 older females for analysis (Fig. 2).

**Fig. 4:**
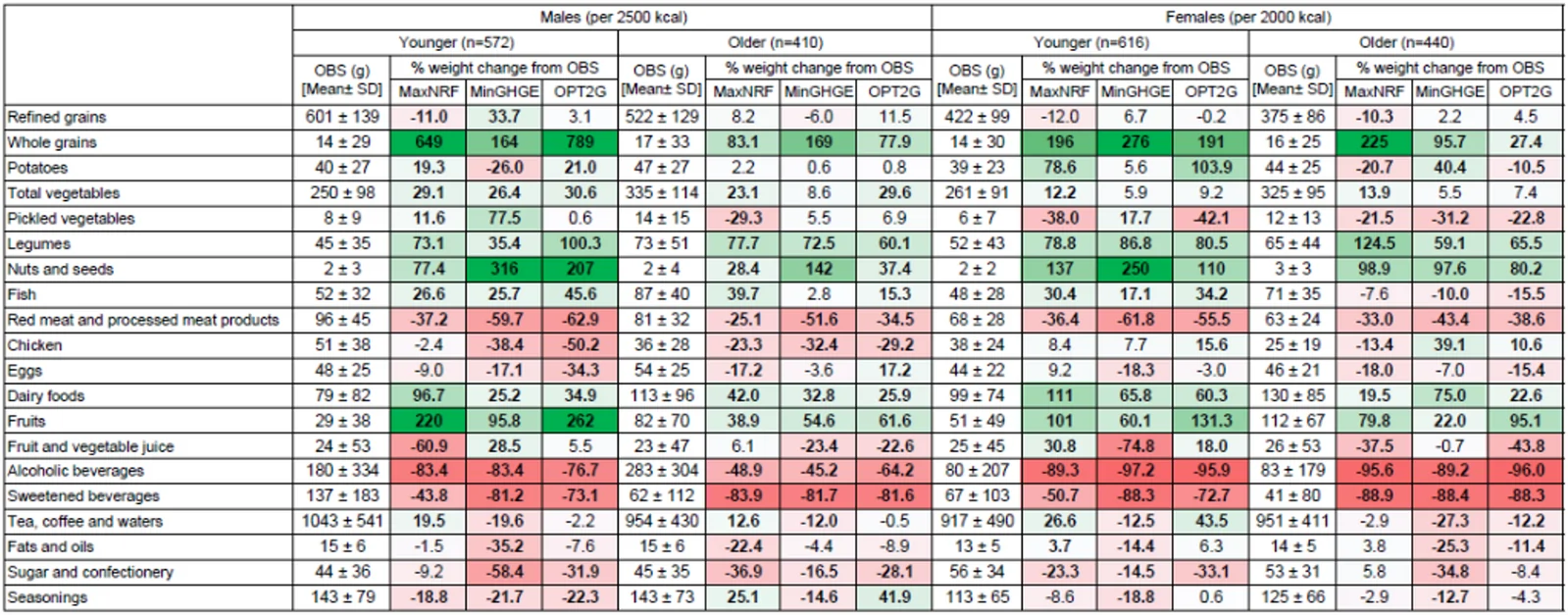
Mean percentage change in food intake from observed to optimised diets among those with inefficient diets. OBS, observed diets; MaxNRF, optimised diets targeted to maximise the Nutrient Rich Food index 9.3 score; MinGHGE, optimised diets targeted to minimise diet-related greenhouse gas emissions; OPT2G, optimised diets which target two goals (i.e., maximise the Nutrient Rich Food index 9.3 score and minimise diet-related greenhouse gas emissions) with the same weight. Participants in the younger groups were aged 18–49 years and those in older groups were aged 50–74 years. Proportion of difference (%) was calculated as [(mean intake in the optimised diets − mean intake in the observed diets) ÷ mean intake in the observed diet] × 100, because individual % difference could not be calculated for those who had zero intake in the observed diet. Values changed by ≥ 10% are written in bold. For visualization purposes, cells are colour-shaded such that positive values are green and negative values are red. Greater intensity of colour reflects larger absolute values. Those for whom a feasible solution could not be obtained were excluded from the analysis (8 younger and 24 older males and 39 younger and 31 older females)

Figure 4, Supplemental Fig. S1 and Supplemental Tables S8 and S9 show a comparison of food intake between observed and optimised diets for those with inefficient diets. In general, all optimised diets showed higher intakes of fruits, unrefined grains, legumes, and dairy foods (> 19%) and lower intakes of red and processed meat products, and alcoholic and sweetened beverages (> 25%) compared with observed diets, although the magnitude of change varied by optimisation model and sex-age group. The proportion of weight change from the observed diets was generally larger in younger than in older males and females. For example, 7–9 food groups required >50% weight changes (compared with observed diets) in all models in younger adults, versus only 3–6 food groups in older adults. Food groups requiring >50% changes in almost all optimised diets (≥ 2 models) across sex–age groups included whole grains, legumes, and sweetened beverages. Additionally, >50% changes were required for nuts and seeds, fruits, and alcoholic beverages in almost all optimised diets among younger males and females.

In addition to the food groups listed above, model-specific trends were observed as follows. For the **MaxNRF** model, these included increased intakes of potatoes (younger males and females), total vegetables, and fish (except older females); and decreased intakes of refined grains (except older males), eggs (older males and females), and fruit/vegetable juice (younger males and older females). For the **MinGHGE** model, these included further reductions in chicken (males), eggs (younger males and females), fruit/vegetable juice (younger females and older males), tea, coffee, water, sugar/confectionery, fats/oils (except younger females), and seasonings. For the **OPT2G** model, these included further increases in potatoes (younger males and females), total vegetables (males), and fish (except older females); and further decreases in pickled vegetables (females), chicken (males), eggs (younger males and older females), fruit/vegetable juice (older males and females), and sugar/confectionery (except older females).

### Nutrient intake quality and environmental indicators in the optimised diets

**Fig. 5:**
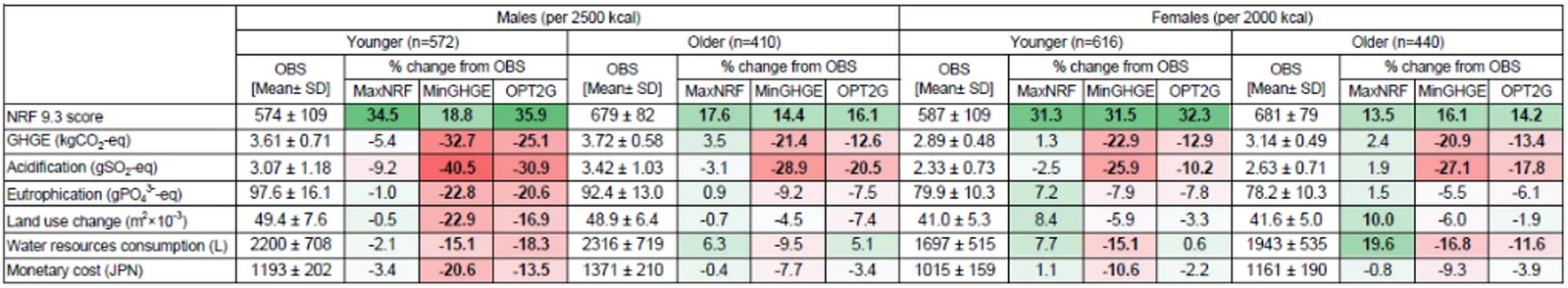
Mean percentage change in NRF9.3 score, environmental impacts, and monetary cost from the observed to optimised diets among those with inefficient diets. GHGE, greenhouse gas emissions; MaxNRF, optimised diets targeted to maximise the Nutrient Rich Food index 9.3; MinGHGE, optimised diets targeted to minimise diet-related greenhouse gas emissions; NRF, Nutrient Rich Food Index; OBS, observed diets; OPT2G, optimised diets which target two goals (i.e., maximise the Nutrient Rich Food index 9.3. and minimise diet-related greenhouse gas emissions) with the same weight. Participants in the younger groups were aged 18–49 years and those in the older groups were aged 50–74 years. Proportion of difference (%) was calculated at each individual level as [(value in the optimised diets − value intake in the observed diets) ÷ value in the observed diet] × 100, and then the mean was calculated across individuals within each sex-age group. Values changed by ≥ 10% are written in bold. For visualisation purposes, cells are colour-shaded such that positive values are green and negative values are red. Greater intensity of colour reflects larger absolute values. Those for whom a feasible solution could not be obtained were excluded from the analysis (8 younger and 24 older males and 39 younger and 31 older females)

All three optimised diets showed NRF 9.3 scores 13.5–35.9% higher than those observed in all sex-age groups (Fig. 5 and Supplemental Tables S10 and S11). In younger males and females, >30% differences were observed across all optimised models, except in the MinGHGE model for younger males (18.8%). In older males and females, an increase in NRF9.3 was similar across models, at around 15%.

Diet-related environmental impact was relatively stable in the MaxNRF model in all sex-age groups. In contrast, optimised diets with the MinGHGE and OPT2G models showed reduced diet-related GHGE by 20.9–32.7% and 12.6–25.1%, respectively, and reduced diet-related acidification by 25.9–40.5% and 12.6–30.9%, respectively, compared with observed diets. Diet-related water resources consumption was reduced by 9.5–16.8% in optimised diets with the MinGHGE model. Diet-related eutrophication and land-use change were reduced by 16.9–22.9% in optimised diets with the MinGHGE and OPT2G models from observed diets in younger males, while showing smaller reductions or no significant difference in other sex-age groups.

Monetary cost remained similar or slightly decreased in MaxNRF, whereas it decreased by 7.7–20.6% in the MinGHGE model and 2.2–13.5% in the OPT2G model.

### Nutrient intake inadequacy in the optimised diets

Overall, the prevalence of inadequate nutrient intake was lower in optimised diets compared with observed diets across all sex-age groups, with some exceptions (Table 3 for males and Table 4 for females). In younger males, the prevalence of inadequacy was higher for macronutrient distributions (e.g., % energy from protein, fat, and carbohydrate) and for vitamin A, C, and riboflavin in the optimised diets with the MinGHGE and OPT2G models in comparison with the observed diets. In older males, the prevalence of inadequate thiamine intake was higher in the MinGHGE model than in the observed diets. In all models and groups, the prevalence of inadequate (i.e., too high) sodium intake remained unchanged at around 85–91% across all diet scenarios. For total fibre, the prevalence of inadequate intake in three optimised diets was lower compared with observed diets, but remained over 60% in younger and older males and younger females, and above 40% in older females. In females, the prevalence of inadequate saturated fat intake remained unchanged or worsened with optimised diets under the MaxNRF and MinGHGE models. In the OPT2G model, it was slightly lower than the observed value but still more than 60%. Nutrient intakes with each optimised diet are shown in Supplemental Tables S12 and S13.

**Table 3:** Number of males with inadequate nutrient intake in observed diets and optimised diets among those with inefficient diets and a feasible solution (per 2500 kcal)

	Younger males (aged 18–49 years; n=572)												Older males (aged 50–74 years; n=410)											
	Observed diets			MaxNRF*			MinGHGE†			OPT2G‡			Observed diets			MaxNRF*			MinGHGE†			OPT2G‡		
	N	(%)		N	(%)		N	(%)		N	(%)		N	(%)		N	(%)		N	(%)		N	(%)	
Protein (< DG)	194	(31)	a	61	(10)	b	340	(54)	c	283	(45)	d	186	(39)	a	30	(6)	b	215	(46)	a	108	(23)	c
Protein (> DG)	4	(1)		0	(0)		0	(0)		0	(0)		1	(0)		0	(0)		0	(0)		0	(0)	
Total fat (< DG)	15	(2)	a	8	(1)	a	200	(32)	b	208	(33)	b	19	(4)	a	1	(0)	b	8	(2)	ac	4	(1)	bc
Total fat (> DG)	257	(41)	a	88	(14)	b	15	(2)	c	14	(2)	c	131	(28)	a	32	(7)	b	49	(10)	b	34	(7)	b
Saturated fat	464	(74)	a	313	(50)	b	111	(18)	c	101	(16)	c	273	(58)	a	172	(37)	b	127	(27)	c	130	(28)	c
Carbohydrates (< DG)	197	(31)	a	20	(3)	b	1	(0)	c	1	(0)	c	194	(41)	a	77	(16)	b	213	(45)	a	46	(10)	c
Carbohydrates (> DG)	8	(1)	a	15	(2)	a	190	(30)	b	230	(37)	c	6	(1)		0	(0)		0	(0)		0	(0)	
Total fibre	568	(91)	a	489	(78)	b	503	(80)	b	391	(62)	c	374	(79)	a	322	(68)	b	336	(71)	bc	349	(74)	c
Sodium§	569	(91)		569	(91)		569	(91)		569	(91)		410	(87)		410	(87)		410	(87)		410	(87)	
Potassium	466	(74)	a	197	(31)	b	479	(77)	a	290	(46)	c	182	(39)	a	7	(1)	b	136	(29)	c	40	(8)	d
*Nutrients with EAR*																								
Protein§	1	(0)		0	(0)		0	(0)		0	(0)		0	(0)		0	(0)		0	(0)		0	(0)	
Vitamin A	455	(73)	ab	427	(68)	a	549	(88)	c	466	(74)	b	263	(56)	a	243	(52)	a	229	(49)	b	202	(43)	b
Thiamine	7	(1)	a	0	(0)	b	0	(0)	b	0	(0)	b	2	(0)	a	0	(0)	a	35	(7)	b	2	(0)	a
Riboflavin	259	(41)	a	158	(25)	b	411	(66)	c	400	(64)	c	77	(16)	a	0	(0)	b	66	(14)	a	27	(6)	c
Niacin§	0	(0)		0	(0)		0	(0)		0	(0)		0	(0)		0	(0)		0	(0)		0	(0)	
Vitamin B_6_	127	(20)	a	8	(1)	b	90	(14)	c	20	(3)	d	24	(5)	a	0	(0)	b	18	(4)	a	6	(1)	b
Folate	29	(5)	a	0	(0)	b	0	(0)	b	0	(0)	b	1	(0)		0	(0)		0	(0)		0	(0)	
Vitamin C	205	(33)	a	39	(6)	b	312	(50)	c	111	(18)	d	60	(13)	a	1	(0)	b	2	(0)	b	1	(0)	b
Calcium	494	(79)	a	215	(34)	b	476	(76)	a	247	(39)	b	221	(47)	a	84	(18)	b	129	(27)	c	91	(19)	b
Magnesium	417	(67)	a	105	(17)	b	310	(50)	c	117	(19)	b	114	(24)	a	1	(0)	b	65	(14)	c	17	(4)	d
Iron	18	(3)	a	0	(0)	b	0	(0)	b	0	(0)	b	3	(1)		0	(0)		0	(0)		0	(0)	
Zinc	23	(4)	a	0	(0)	b	0	(0)	b	0	(0)	b	16	(3)	a	0	(0)	b	31	(7)	a	1	(0)	b
*Nutrients with WHO’s recommendation*																								
Added sugar	280	(45)	a	192	(31)	b	52	(8)	c	59	(9)	c	155	(33)	a	29	(6)	b	101	(21)	c	74	(16)	d

DG, Tentative Dietary Goal for Preventing Lifestyle-related Diseases; EAR, Estimated Average Requirement; WHO, World Health Organisation

Values with different letters within a row (a,b,c,d) are significantly different according to pairwise McNemar’s tests (two-sided *P*< 0.0083 [= 0.05/6]). Values sharing the same letter are not significantly different

^*^Optimised diets targeted to maximise the Nutrient Rich Food index 9.3 score

^†^Optimised diets targeted to minimise diet-related greenhouse gas emissions

^‡^Optimised diets targeted to achieve two goals (i.e., maximise the Nutrient Rich Food index 9.3 score, and minimise diet-related greenhouse gas emissions) with the same weight

^§^McNemar’s test was not conducted

**Table 4:** Number of females with inadequate nutrient intake in observed diets and optimised diets among those with inefficient diets and a feasible solution (per 2000 kcal)

	Younger females (aged 18–49 years; n=616)									Older females (aged 50–74 years; n=440)											
	Observed diets			MaxNRF*			OPT2G‡			Observed diets			MaxNRF*			MinGHGE†			OPT2G‡		
	N	(%)		N	(%)		N	(%)		N	(%)		N	(%)		N	(%)		N	(%)	
*Nutrients with DG*																					
Protein (< DG)	114	(16)	a	8	(1)	b	19	(3)	c	132	(27)	a	100	(21)	b	50	(10)	b	155	(32)	c
Protein (> DG)	1	(0)		0	(0)		0	(0)		5	(1)		2	(0)		2	(0)		2	(0)	
Total fat (< DG)	8	(1)	a	0	(0)	b	0	(0)	b	2	(0)		0	(0)		0	(0)		0	(0)	
Total fat (> DG)	353	(49)	a	353	(49)	a	158	(22)	b	223	(46)	a	149	(31)	b	116	(24)	b	81	(17)	c
Saturated fat	555	(77)	a	556	(77)	a	459	(63)	b	390	(80)	a	386	(79)	a	426	(88)	b	354	(73)	c
Carbohydrates (< DG)	202	(28)	a	141	(19)	b	15	(2)	c	153	(31)	a	31	(6)	b	12	(2)	c	16	(3)	c
Carbohydrates (> DG)	5	(1)	a	0	(0)	b	0	(0)	b	0	(0)		0	(0)		0	(0)		0	(0)	
Total fibre	591	(82)	a	458	(63)	b	457	(63)	b	357	(73)	a	242	(50)	b	226	(47)	b	192	(40)	c
Sodium§	616	(85)		615	(85)		616	(85)		440	(91)		440	(91)		440	(91)		440	(91)	
Potassium	409	(56)	a	90	(12)	b	135	(19)	d	120	(25)	a	3	(1)	b	8	(2)	b	25	(5)	c
*Nutrients with EAR*																					
Protein§	0	(0)		0	(0)		0	(0)		0	(0)		0	(0)		0	(0)		0	(0)	
Vitamin A	329	(45)	a	48	(7)	b	48	(7)	b	144	(30)	a	6	(1)	b	10	(2)	b	5	(1)	b
Thiamine	3	(0)		0	(0)		0	(0)		0	(0)		0	(0)		0	(0)		0	(0)	
Riboflavin	83	(11)	a	3	(0)	b	13	(2)	c	7	(1)	a	0	(0)	b	0	(0)	b	0	(0)	b
Niacin§	0	(0)		0	(0)		0	(0)		0	(0)		0	(0)		0	(0)		0	(0)	
Vitamin B_6_	96	(13)	a	3	(0)	b	3	(0)	b	13	(3)	a	0	(0)	b	0	(0)	b	0	(0)	b
Folate	32	(4)	a	0	(0)	b	0	(0)	b	0	(0)		0	(0)		0	(0)		0	(0)	
Vitamin C	202	(28)	a	25	(3)	b	15	(2)	c	32	(7)	a	0	(0)	b	12	(2)	c	0	(0)	b
Calcium	434	(60)	a	103	(14)	b	237	(33)	d	183	(38)	a	23	(5)	b	18	(4)	b	90	(19)	c
Magnesium	242	(33)	a	24	(3)	b	31	(4)	b	55	(11)	a	0	(0)	b	3	(1)	b	6	(1)	b
Iron	276	(38)	a	64	(9)	b	105	(14)	d	63	(13)	a	0	(0)	b	25	(5)	c	28	(6)	c
Zinc	12	(2)	a	0	(0)	b	0	(0)	b	0	(0)		0	(0)		0	(0)		0	(0)	
*Nutrients with WHO’s recommendation*																					
Added sugar (g)	414	(57)	a	314	(43)	b	208	(29)	c	279	(57)	a	303	(62)	a	167	(34)	b	283	(58)	a

DG, Tentative Dietary Goal for Preventing Lifestyle-related Diseases; EAR, Estimated Average Requirement; WHO, World Health Organisation

Values with different letters within a row (a,b,c,d) are significantly different according to pairwise McNemar’s tests (two-sided *P*< 0.0083 [= 0.05/6]). Values sharing the same letter are not significantly different. Those for whom a feasible solution could not be obtained were excluded from the analysis (39 younger female and 31 older female participants)

^*^Optimised diets targeted to maximise the Nutrient Rich Food index 9.3 score

^†^Optimised diets targeted to minimise diet-related greenhouse gas emissions

^‡^Optimised diets targeted to achieve two goals (i.e., maximise the Nutrient Rich Food index 9.3 score and minimise diet-related greenhouse gas emissions) with the same weight

^§^McNemar’s test was not conducted

## Discussion

In this study, we found that NRF9.3 scores were 13–35% higher and the prevalence of nutrient inadequacy was generally lower (except for younger males in the MinGHGE model) in all three optimised diets and across all sex-age groups than in the observed diets, indicating better overall nutrient quality. The difference between optimised and observed diets was particularly larger among younger males and females (7–9 food groups) than among older groups (3–6 food groups); in other words, more food groups required>50% weight changes relative to the observed diets. However, environmental indicators tended to be stable unless the model specifically aimed to minimise diet-related GHGE (i.e., the MinGHGE and OPT2G models). Conversely, in younger males, optimised diets targeting minimisation of diet-related GHGE showed poorer macronutrient balance and a greater prevalence of inadequate intakes of vitamin A, vitamin C, and riboflavin than observed diets.

The differences between optimised and observed diets were generally larger among younger males and females than among the older adults for NRF 9.3 scores, environmental indicators, and diet composition. A previous study that explored optimised diets for Japanese adults meeting the nutrient recommendations of the DRIs using linear programming reported similar results [14]. In that study, the amount by which young adults needed to increase their fruit and vegetable intake (> 50% of observed diets) was substantially greater than that required for older adults (< 10%). The reason why younger adults required larger modifications was unknown, but might be due to differences in dietary habits between age groups and lower variation in dietary intake among older adults. In our present study, mean NRF9.3 score was higher in older adults with inefficient diets than in younger adults with efficient diets in both sexes. This suggests that older adults already had healthier dietary habits than younger adults, as shown in other studies [11]. Since few studies have explored optimised diets by age group, comparing our results with other studies is difficult.

In younger males, optimised diets targeting minimisation of diet-related GHGE showed higher carbohydrate intake and lower protein and fat intake. It has been widely reported that plant-based staple foods, such as grains, generally have lower diet-related GHGE than animal-based foods, partly because livestock production requires additional inputs [31, 41, 42]. Because younger males have a relatively higher intake of refined grains than other sex-age groups, an optimised diet that reduces diet-related GHGE might lead to a much higher cereal intake. Additionally, optimised diets by the MinGHGE model showed a greater prevalence of inadequate intakes of vitamin A, vitamin C, and riboflavin than in the observed and other scenario diets. In Japan, major dietary sources of riboflavin are meat, fish, eggs, and dairy products [10]. Thus, larger reductions in meat intake from observed diets might lead to inadequate riboflavin intake. For vitamin A, the major dietary sources are vegetables, followed by animal-based foods. For vitamin C, vegetables and fruits are major sources [10]. It is possible that, among some participants, a dietary pattern emerged in which intake of refined grains, such as white rice, increased markedly relative to their observed diets, while consumption of vegetables and fruits increased only minimally. Such diets may have contributed to deficiencies in vitamins A and C. Considered together, these findings indicate the need for caution with some vitamins when diet-related GHGE minimisation is targeted in younger males.

The prevalence of exceeding the DG for sodium intake did not differ between the three optimised and observed diets in all sex-age groups (namely, 85–91%), and that of saturated fat remained over 60% in females across all models. For these nutrients, exceeding the DG upper intake limit is prevalent [11]. For sodium, intake in those with efficient diets was less than in those with inefficient diets, except among older males, although the mean intake remained above the DG. In a population with high intakes of sodium and saturated fat, optimised diets derived as linear combinations of the observed diets are also likely to retain relatively high intakes of these nutrients. In addition, previous studies from Japan have reported a positive association of sodium intake with healthy dietary patterns [35, 37]. For saturated fat, no significant difference was observed between efficient and inefficient diets among females. Taken together, these findings suggest that the achievement of better diets through the DEA-diet model is difficult for nutrients with a high prevalence of excessive intake in observed diets.

The optimised diets with the MinGHGE and OPT2G models had 20.9–32.7% and 12.6–25.1% lower diet-related GHGE, respectively, than the observed diets, with greater reductions observed in younger males than in other sex-age groups. The greater reductions in GHGE among younger males may partly reflect the greater variation in baseline diet-related GHGE among efficient diets in this group, which provided more scope to identify lower-GHGE diets through benchmarking. In addition, their higher baseline intakes of dietary components to decrease may have allowed larger reductions in these food groups during optimisation, contributing to the greater reductions in GHGE. These differences between optimised and observed diets were comparable to or greater than those reported previously using the DEA-diet model. Among 369 Japanese adults (aged 20–69 years), differences of 16% in males and 12% in females were found [23]. Among adults in four European countries, 13–28% lower diet-related GHGE was obtained than observed diets [21]. A study in China reported 20–35% lower diet-related GHGE than observed [24]. The larger sample size in the present study may have enabled us to obtain dietary patterns with greater improvements. In addition, the previous study calculated changes from observed diets using the total sample, including those with efficient diets, on the assumption that their dietary intake was the same as in the observed diets [23]. This method may underestimate the reduction reported in the previous study. Although the reductions observed here were smaller than those from linear programming approaches (> 50%) [15, 16], the optimised diets identified in this study might be a first step in dietary change toward sustainable diets. Nevertheless, even in models that considered diet-related GHGE minimisation, eutrophication and land use change remained almost unchanged. These findings suggest that, even though correlations between GHGE and other environmental impact indicators have been reported, minimising GHGE through dietary optimisation does not necessarily result in similarly large reductions in other environmental impacts. Therefore, future research will be required to incorporate additional environmental indicators related to food production beyond GHGE, such as acidification and land use change, into the model.

Our results provide preliminary insight that may inform the development of future FBDGs for the Japanese population. Across sex-age groups the optimised diets generally suggested lower intakes of alcoholic and sweetened beverages, and red and processed meat products, and higher intakes of whole grains, legumes, nuts and seeds, and fruits. In addition, among females, the OPT2G model showed the potential to simultaneously improve several aspects of nutritional quality while reducing diet-related GHGE. However, these findings should be interpreted cautiously given the non-representativeness of the study population, the use of food price data collected more than 12 years ago [34], and the inability of the optimisation models to resolve all nutrient inadequacies. In particular, although the OPT2G model improved several aspects of diet quality among females, a substantial proportion still exceeded the saturated fat limit. Among males, some nutrient inadequacies were more prevalent in the optimised than in the observed diets. Thus, the optimised diets identified in this study should not be considered direct recommendations for future FBDGs, but rather as potential dietary patterns that warrant further investigation. Future optimisation studies using more representative and up-to-date data and explicitly incorporating nutritional adequacy constraints are needed to identify dietary patterns that are nutritionally adequate, environmentally sustainable, affordable, and culturally acceptable for the Japanese population.

This study should be interpreted in light of several limitations. First, our participants were not randomly selected from a representative sample of the general Japanese population, but were volunteers. Accordingly, they might be somewhat more health-conscious and have healthier dietary habits than the general population. In addition, our participants had higher education levels than the nationally representative sample aged 20–74 years (43.2% for junior or senior high school, 15.6% for 2-year college or technical school, 24.2% for university or higher; education level reported in the Population Census of Japan 2020 [43]). Second, when evaluating nutrient intake adequacy, day-to-day variation is not accounted for, which may have led to an overestimation of the proportions of inadequate nutrient intake. Moreover, energy intake was standardised to 2500 kcal in males and 2000 kcal in females. Thus, the prevalence of nutrient intake inadequacy should be interpreted with caution. However, in the present study, the purpose was not to evaluate nutrient intake inadequacy per se, but rather to examine how it changes relative to an observed diet. In addition, the use of 8-day dietary records collected across all four seasons provided dietary intake data that reflected both day-to-day and seasonal variations as comprehensively as possible. Third, the diet-related environmental indicators might have been underestimated due to the exclusion of emissions from post-production phases. In addition, the number of food items included in IDEA version 3.4.1 is approximately 350, which is far fewer than those listed in the Standard Tables of Food Composition in Japan. Furthermore, the food classification scheme used in IDEA also differs from that of the Standard Tables of Food Composition in Japan. Consequently, some foods may not be evaluated accurately with respect to their environmental indicators. Moreover, IDEA assumes domestic production in Japan. Although an international version of the dataset is available, only the electricity mix is recalculated using values from other countries, and the actual food production processes in those countries are not appropriately reflected. Given that Japan imports a large proportion of its food [44], it is necessary to develop a database that accounts for international trade and reflects these values. Fourth, NRF 9.3 was originally developed using US-based reference values and was not originally designed or validated for Japanese populations. However, its applicability to the Japanese population has been examined [29], and previous studies have used Japanese DRIs as RDVs in NRF 9.3 [28, 45], with one study reporting an association with lower mortality among Japanese adults [45]. Fifth, the food price data were from the National Retail Price Survey 2013, which is over 12 years old. Food prices have increased substantially since 2013 [46], and the magnitude of price changes may have differed across food groups and individual food items [47]. Although the cost constraint was defined relative to each individual’s observed dietary cost, such differential price changes could have affected the composition of the optimised diets. Sixth, potential selection bias may have been introduced by the exclusion of 102 participants for whom no feasible solution could be obtained. Participants without feasible solutions, particularly younger females, tended to have lower monetary costs for their observed diets. Their exclusion may therefore have led to an overestimation of the affordability and feasibility of the optimised diets.

## Conclusions

This study calculated alternative diets that have greater nutritional value, lower diet-related GHGE, and remain affordable. When nutrient intake quality was maximised, nutrient intake inadequacy was minimised across all sex–age groups, whereas environmental impacts remained almost unchanged relative to observed diets. When diet-related GHGE was minimised, a lower environmental impact than observed was shown across all sex-age groups; however, in younger males, macronutrient balance and the adequacy of several vitamins were less favourable. In the other sex–age groups, no nutrients showed worsening inadequacy. In all models, younger individuals tended to show greater improvements than older individuals, which may partly reflect their less favourable baseline dietary intake. Similar trends were observed in the model that considered both nutrient intake quality and diet-related GHGE. Thus, the DEA-diet model can enhance nutrient adequacy and reduce environmental impact, although attention should be paid to certain nutrients when minimising diet-related GHGE. These findings suggest that dietary changes among younger generations have greater potential to improve both nutritional quality and environmental impact, assuming similar adoption of the optimised dietary changes across age groups.

## Supplementary Information

Below is the link to the electronic supplementary material.

Supplementary Material 1

Supplementary Material 2

## Data Availability

The datasets generated and analysed during the present study are not publicly available due to restrictions imposed by the Consumer Affairs Agency, Japan.
