# Supplementary material for: Optimizing Japanese dietary patterns to improve nutritional quality, reduce environmental impact under affordability constraints in younger and older adults from the nationwide MINNADE study
Source: Eur J Nutr. 2026 Oct 13;65(8):282. doi: 10.1007/s00394-026-04129-x (PMC13661754; doi:10.1007/s00394-026-04129-x)
Supplement: Supplementary file 1 [file 394_2026_4129_MOESM1_ESM.docx]

# Supplemental Table S1. Food group classification used in this study

| Food group | Subgroup | Main food items |
| --- | --- | --- |
| Refined grain |  | Well-milled rice, oats, bread, noodles, and flour |
| Whole grain |  | Brown rice, whole grain flour, whole barley flour, whole grain noodle, 70% milled rice, half milled rice |
| Potatoes |  | Potatoes, sweet potatoes, Japanese yam, French fries, starch |
| Total vegetables (excluding pickled vegetables) | | Dark green vegetables, tomatoes, orange vegetables, other vegetables, mushrooms, and seaweeds, excluding pickled vegetables |
| Pickled vegetables |  | *Takuan-zuke*, *umeboshi*, *nara-zuke*, kimchi, ginger pickles |
| Legumes |  | *Azuki* beans, kidney beans, peas, soybeans and their products (tofu, *natto*) |
| Nuts and seeds |  | Almonds, chestnuts, walnuts, peanuts |
| Fish | Oily fish and lean fish | Salmon, Japanese pilchard, pacific saury, mackerel, eel, horse mackerel, fatty tuna, yellowtail, atka mackerel, sea bream, lean tuna, codfish, marlin fish, fish roe, excluding canned fish |
|  | Other shellfish and processed seafood | Canned tuna, dried fish, clams, scallops, whelk, octopus, squid, shrimp, crab, processed seafood, bonito flakes, dried sardines, sea urchins |
| Red meat and processed meat products |  | Pork, beef, mutton, horse (including organ), ham, sausage, bacon |
| Chicken |  | Chicken, duck (including offal), other meat |
| Eggs |  | Chicken eggs, quail eggs |
| Dairy foods |  | Ordinary liquid milk, whole milk powder, cream, yoghurt, natural cheese, processed cheese |
| Fruits |  | Strawberries, banana, apple, orange, grapefruit, grapes, kiwifruit, watermelon, dried fruits |
| Fruit and vegetable juice |  | 100% fruit and vegetable juices |
| Alcoholic beverages |  | Beer, wine, sake, whiskey, mirin (a type of sake for cooking) |
| Sweetened beverages |  | 50% fruit juice beverage (Valencia orange, pineapple), lactic acid bacteria beverages, fruit-flavoured and coloured drink, cola drink |
| Tea, coffee and waters |  | Green teas, oolong tea, black tea, coffee, cocoa, water |
| Fats and oils |  | Lard, butter, olive oil, sesame oil, rapeseed oil, margarine, shortening, mayonnaise |
| Sugar and confectionery |  | Brown sugar, sugar, honey, jams, *manju* (baked or steamed dough stuffed with filling), drops, rice crackers, bean jam bun, sponge cake, doughnuts, biscuits, milk chocolate, chewing gums, ice cream |
| Seasonings |  | Worcester sauces, soy sauces, common salt, vinegars, soup stocks, dressings, miso, roux |

# Supplemental Table S2. Sex- and age-specific reference daily values used in calculation of the Nutrient-Rich Food (NRF) 9.3 score*

|  | Males | | | |  | Females | | | |
| --- | --- | --- | --- | --- | --- | --- | --- | --- | --- |
|  | 18–29 years | 30–49 years | 50–64 years | 65–74 years |  | 18–29 years | 30–49 years | 50–64 years | 65–74 years |
| Energy (kcal) | 2500 | 2500 | 2500 | 2500 |  | 2000 | 2000 | 2000 | 2000 |
| Qualifying nutrients |  |  |  |  |  |  |  |  |  |
| Protein (g)† | 65 | 65 | 65 | 60 |  | 50 | 50 | 50 | 50 |
| Total dietary fibre (g)‡ | 20 | 22 | 22 | 21 |  | 18 | 18 | 18 | 18 |
| Vitamin A (μg RAE)† | 850 | 900 | 900 | 850 |  | 650 | 700 | 700 | 700 |
| Vitamin C (mg)† | 100 | 100 | 100 | 100 |  | 100 | 100 | 100 | 100 |
| Vitamin D (μg)‡ | 9 | 9 | 9 | 9 |  | 9 | 9 | 9 | 9 |
| Calcium (mg)† | 800 | 750 | 750 | 750 |  | 650 | 650 | 650 | 650 |
| Iron (mg)† | 7 | 7.5 | 7 | 7 |  | 10 | 10.5 | 10.5 | 6 |
| Potassium (mg)§ | 2500 | 2500 | 2500 | 2500 |  | 2000 | 2000 | 2000 | 2000 |
| Magnesium (mg)† | 340 | 380 | 370 | 350 |  | 280 | 290 | 290 | 280 |
| Disqualifying nutrients |  |  |  |  |  |  |  |  |  |
| Saturated fat (%energy)† | <7 | <7 | <7 | <7 |  | <7 | <7 | <7 | <7 |
| Sodium (g NaCl equivalent/day)‡ | <7.5 | <7.5 | <7.5 | <7.5 |  | <6.5 | <6.5 | <6.5 | <6.5 |
| Added sugar (%energy)\|\| | <5 | <5 | <5 | <5 |  | <5 | <5 | <5 | <5 |

RAE, Retinol Activity Equivalents.

*Values were derived from the Dietary Reference Intakes for Japanese, 2025 except for added sugar.

†Recommended Dietary Allowance, defined as “an estimate of the daily average dietary intake that satisfies the needs of most of the individuals belonging to a population (97–98%), on the basis of the distribution of the measured requirements of a study population.

‡Tentative Dietary Goal for Preventing Lifestyle-related Diseases, defined as “the average daily-nutrient-intake level (or ranges) that Japanese should currently aim to consume primarily to prevent chronic diseases

§Adequate Intake, defined as “a recommended average daily-nutrient-intake level based on observed or experimentally determined approximations or estimates of nutrient intake by a group (or groups) of apparently healthy people that are assumed to be adequate”.

||Determined based on the World Health Organization’s conditional recommendation (5% of energy)

# Supplemental Table S3. Sex- and age-specific reference daily values of Dietary Reference Intakes for Japanese 2025

|  | Males | | | |  | Females | | | |
| --- | --- | --- | --- | --- | --- | --- | --- | --- | --- |
|  | 18–29 years | 30–49 years | 50–64 years | 65–74 years |  | 18–29 years | 30–49 years | 50–64 years | 65–74 years |
| Nutrients with DG* |  |  |  |  |  |  |  |  |  |
| Protein (%E) | 13–20 | 13–20 | 14–20 | 15–20 |  | 13–20 | 13–20 | 14–20 | 15–20 |
| Total fat (%E) | 20–30 | 20–30 | 20–30 | 20–30 |  | 20–30 | 20–30 | 20–30 | 20–30 |
| Saturated fat (%E) | <7 | <7 | <7 | <7 |  | <7 | <7 | <7 | <7 |
| Carbohydrates (%E) | 50–65 | 50–65 | 50–65 | 50–65 |  | 50–65 | 50–65 | 50–65 | 50–65 |
| Total fibre (g) | 20 | 22 | 22 | 21 |  | 18 | 18 | 18 | 17 |
| Sodium (g salt) | <7.5 | <7.5 | <7.5 | <7.5 |  | <6.5 | <6.5 | <6.5 | <6.5 |
| Potassium (mg) | 3000 | 3000 | 3000 | 3000 |  | 2600 | 2600 | 2600 | 2600 |
| Nutrients with EAR† |  |  |  |  |  |  |  |  |  |
| Protein (g) | 50 | 50 | 50 | 50 |  | 40 | 40 | 40 | 40 |
| Vitamin A (µgRAE) | 600 | 650 | 650 | 600 |  | 450 | 500 | 500 | 500 |
| Thiamine (mg) | 0.8 | 0.8 | 0.8 | 0.7 |  | 0.6 | 0.6 | 0.6 | 0.6 |
| Riboflavin (mg) | 1.3 | 1.4 | 1.3 | 1.2 |  | 1 | 1 | 1 | 0.9 |
| Niacin (mg) | 13 | 13 | 13 | 11 |  | 9 | 10 | 9 | 9 |
| Vitamin B_6_ (mg) | 1.2 | 1.2 | 1.2 | 1.2 |  | 1 | 1 | 1 | 1 |
| Folate (μg) | 200 | 200 | 200 | 200 |  | 200 | 200 | 200 | 200 |
| Vitamin C (mg) | 80 | 80 | 80 | 80 |  | 80 | 80 | 80 | 80 |
| Calcium (mg) | 650 | 650 | 650 | 600 |  | 550 | 550 | 550 | 550 |
| Magnesium (mg) | 280 | 320 | 310 | 290 |  | 230 | 240 | 240 | 240 |
| Iron (mg) | 5.5 | 6 | 6 | 5.5 |  | 7 | 7.5 | 7.5 | 5 |
| Zinc (mg) | 7.5 | 8 | 8 | 7.5 |  | 6 | 6.5 | 6.5 | 6.5 |

DG, Tentative Dietary Goal for Preventing Lifestyle-related Diseases; EAR, Estimated Average Requirement; RAE, Retinol Activity Equivalents; %E, percentage of total energy intake.

*Tentative Dietary Goal for Preventing Lifestyle-related Diseases, defined as “the average daily-nutrient-intake level (or ranges) that Japanese should currently aim to consume primarily to prevent chronic diseases”

†The Estimated Average Requirement is defined as “the estimated intake amount that meets the requirements of 50% of the individuals belonging to an age or sex group."

# Supplemental Table S4. Comparison of food and nutrient intakes between males with efficient diets and inefficient diets (per 2500 kcal).*

|  | Younger | | | | | |  | Older | | | | | |
| --- | --- | --- | --- | --- | --- | --- | --- | --- | --- | --- | --- | --- | --- |
|  | Efficient diets (n=131) | |  | Inefficient diets (n=580) | | P-value† |  | Efficient diets (n=131) | |  | Inefficient diets (n=434) | | P-value† |
|  | Mean | SD |  | Mean | SD |  |  | Mean | SD |  | Mean | SD |  |
| Food intake |  |  |  |  |  |  |  |  |  |  |  |  |  |
| Refined cereals | 548.5 | 172.9 |  | 602.6 | 139.6 | <0.0001 |  | 465.7 | 167.7 |  | 526.3 | 131.7 | <0.0001 |
| Whole grain‡ | 46.4 | 87.1 |  | 14.2 | 29.3 | 0.0001 |  | 55.8 | 90.7 |  | 17.8 | 32.9 | <0.0001 |
| Potatoes | 40.0 | 28.6 |  | 40.1 | 26.8 | <0.0001 |  | 49.5 | 30.4 |  | 46.1 | 26.6 | 0.21 |
| Total vegetables (excluding pickled vegetables)‡ | 309.8 | 140.3 |  | 249.6 | 99.3 | 1.0 |  | 407.5 | 159.3 |  | 334.4 | 115.4 | <0.0001 |
| Green and yellow vegetables | 101.4 | 62.2 |  | 69.7 | 38.9 | <0.0001 |  | 132.5 | 75.4 |  | 102.3 | 48.9 | <0.0001 |
| Other vegetables | 181.3 | 82.8 |  | 159.9 | 69.5 | <0.0001 |  | 237.4 | 98.0 |  | 203.6 | 75.5 | <0.0001 |
| Mushrooms | 8.2 | 13.3 |  | 7.7 | 8.6 | 0.002 |  | 14.3 | 17.0 |  | 13.9 | 15.3 | 0.81 |
| Seaweed | 15.1 | 17.2 |  | 11.9 | 11.9 | 0.64 |  | 20.1 | 17.9 |  | 16.1 | 13.7 | 0.0072 |
| Pickled vegetables | 12.0 | 14.7 |  | 8.2 | 9.1 | 0.011 |  | 17.6 | 19.3 |  | 12.3 | 12.7 | 0.0003 |
| Legumes (soy, soy products, other beans and peas) ‡ | 82.8 | 85.1 |  | 45.4 | 36.0 | 0.0001 |  | 100.6 | 75.8 |  | 72.9 | 50.3 | <0.0001 |
| Nuts and seeds‡ | 3.5 | 7.5 |  | 1.8 | 2.7 | <0.0001 |  | 5.1 | 8.1 |  | 2.6 | 3.9 | <0.0001 |
| Oily fish and lean fish‡ | 43.8 | 38.5 |  | 26.1 | 21.8 | <0.0001 |  | 63.4 | 43.9 |  | 43.1 | 27.1 | <0.0001 |
| Other fish, seafoods, and processed seafood | 26.8 | 20.6 |  | 25.6 | 18.6 | <0.0001 |  | 43.0 | 28.5 |  | 42.9 | 25.9 | 1.0 |
| Red meat and processed meat products§ | 81.9 | 44.2 |  | 96.2 | 44.9 | 0.51 |  | 68.2 | 39.0 |  | 81.4 | 32.6 | 0.0001 |
| Chicken | 50.1 | 46.9 |  | 50.8 | 38.2 | 0.001 |  | 27.6 | 25.2 |  | 35.0 | 28.1 | 0.008 |
| Eggs | 46.8 | 30.1 |  | 48.0 | 25.4 | 0.86 |  | 51.6 | 28.8 |  | 54.5 | 25.3 | 0.27 |
| Dairy foods‡ | 158.4 | 148.2 |  | 79.7 | 82.3 | 0.63 |  | 167.9 | 148.2 |  | 114.4 | 99.1 | <0.0001 |
| Fruits‡ | 62.4 | 71.7 |  | 28.8 | 37.5 | <0.0001 |  | 130.1 | 107.4 |  | 81.4 | 71.2 | <0.0001 |
| Fruit and vegetable juice | 27.0 | 56.1 |  | 24.0 | 52.4 | <0.0001 |  | 31.6 | 66.5 |  | 22.4 | 46.3 | 0.076 |
| Alcoholic beverages§ | 88.2 | 246.0 |  | 177.8 | 332.0 | 0.56 |  | 176.0 | 339.7 |  | 270.8 | 301.5 | 0.0023 |
| Sweetened beverages§ | 111.9 | 250.5 |  | 136.0 | 182.5 | 0.004 |  | 25.4 | 58.9 |  | 60.4 | 109.7 | 0.001 |
| Tea, coffee and waters | 1093.9 | 633.9 |  | 1042.6 | 539.3 | 0.21 |  | 1133.0 | 600.4 |  | 949.8 | 427.5 | 0.0001 |
| Fats and oils | 14.9 | 6.6 |  | 15.0 | 6.2 | 0.34 |  | 14.9 | 7.3 |  | 14.8 | 5.8 | 0.92 |
| Sugar and confectionery | 46.7 | 48.4 |  | 44.0 | 36.3 | 0.86 |  | 45.9 | 32.9 |  | 45.7 | 34.9 | 0.95 |
| Seasonings | 136.6 | 79.0 |  | 142.9 | 78.7 | 0.48 |  | 158.1 | 89.5 |  | 143.6 | 73.4 | 0.06 |
| Nutrient intake |  |  |  |  |  |  |  |  |  |  |  |  |  |
| Protein (g) | 93.2 | 16.3 |  | 85.9 | 11.9 | <0.0001 |  | 96.6 | 13.4 |  | 91.5 | 10.1 | <0.0001 |
| Total fat (g) | 80.7 | 15.5 |  | 81.8 | 14.3 | 0.42 |  | 78.7 | 15.4 |  | 76.9 | 12.4 | 0.18 |
| Saturated fat (g) | 23.6 | 6.2 |  | 23.9 | 5.3 | 0.62 |  | 21.8 | 5.7 |  | 21.7 | 4.7 | 0.82 |
| Carbohydrates (g) | 329.7 | 46.3 |  | 325.2 | 39.9 | 0.26 |  | 320.5 | 47.3 |  | 313.8 | 42.2 | 0.12 |
| Total fibre (g) | 15.8 | 4.8 |  | 13.1 | 2.7 | <0.0001 |  | 19.5 | 5.3 |  | 16.1 | 4.3 | <0.0001 |
| Vitamin A (μgRAE) | 619.3 | 349.6 |  | 542.2 | 422.4 | 0.051 |  | 846.0 | 694.9 |  | 666.9 | 506.9 | 0.0013 |
| Vitamin D (μg) | 9.0 | 6.0 |  | 7.2 | 3.6 | <0.0001 |  | 11.7 | 5.3 |  | 10.5 | 4.7 | 0.010 |
| Vitamin E (mg) | 11.1 | 2.6 |  | 10.1 | 2.1 | <0.0001 |  | 12.3 | 3.1 |  | 11.0 | 2.2 | <0.0001 |
| Vitamin K (μg) | 298.1 | 146.2 |  | 234.8 | 95.3 | <0.0001 |  | 369.5 | 163.8 |  | 293.9 | 123.1 | <0.0001 |
| Thiamine (mg) | 1.3 | 0.3 |  | 1.3 | 0.3 | 0.09 |  | 1.3 | 0.3 |  | 1.3 | 0.2 | 0.026 |
| Riboflavin (mg) | 1.6 | 0.3 |  | 1.4 | 0.3 | <0.0001 |  | 1.8 | 0.4 |  | 1.6 | 0.3 | <0.0001 |
| Niacine (mg) | 23.0 | 6.7 |  | 21.3 | 5.1 | 0.001 |  | 25.0 | 6.1 |  | 23.3 | 4.5 | 0.0005 |
| Vitamin B_6_ (mg) | 1.7 | 0.5 |  | 1.4 | 0.3 | <0.0001 |  | 1.9 | 0.4 |  | 1.7 | 0.3 | <0.0001 |
| Vitamin B_12_ (μg) | 7.3 | 4.5 |  | 5.9 | 3.3 | <0.0001 |  | 10.2 | 5.2 |  | 8.4 | 3.9 | <0.0001 |
| Folate (μg) | 397 | 134 |  | 334 | 97 | <0.0001 |  | 502 | 138 |  | 422 | 121 | <0.0001 |
| Pantothenic acid (mg) | 7.4 | 1.5 |  | 6.5 | 1.0 | <0.0001 |  | 7.9 | 1.4 |  | 7.1 | 1.1 | <0.0001 |
| Vitamin C (mg) | 117 | 55 |  | 96 | 39 | <0.0001 |  | 159 | 55 |  | 129 | 48 | <0.0001 |
| Sodium (g salt)§ | 11.4 | 2.6 |  | 12.1 | 2.2 | 0.001 |  | 12.4 | 2.8 |  | 12.8 | 2.2 | 0.11 |
| Potassium (mg) | 3095 | 718 |  | 2613 | 483 | <0.0001 |  | 3654 | 783 |  | 3135 | 600 | <0.0001 |
| Calcium (mg) | 629 | 202 |  | 498 | 137 | <0.0001 |  | 734 | 220 |  | 603 | 156 | <0.0001 |
| Magnesium (mg) | 334 | 78 |  | 278 | 47 | <0.0001 |  | 391 | 76 |  | 334 | 57 | <0.0001 |
| Iron (mg) | 9.8 | 2.7 |  | 8.4 | 1.5 | <0.0001 |  | 11.3 | 2.5 |  | 9.8 | 1.8 | <0.0001 |
| Zinc (mg) | 11.2 | 1.8 |  | 10.5 | 1.7 | 0.0001 |  | 11.1 | 1.7 |  | 10.4 | 1.4 | <0.0001 |
| Copper (mg) | 1.4 | 0.3 |  | 1.3 | 0.2 | <0.0001 |  | 1.6 | 0.3 |  | 1.4 | 0.2 | <0.0001 |
| Alcohol (g) | 6.6 | 17.3 |  | 10.8 | 19.6 | 0.027 |  | 14.5 | 25.3 |  | 21.9 | 24.1 | 0.003 |
| Added sugar (g) | 32.1 | 27.9 |  | 35.6 | 22.7 | 0.13 |  | 28.9 | 15.7 |  | 28.7 | 15.7 | 0.90 |

RAE, Retinol Activity Equivalents.

*Efficient diets were identified separately for 711 younger males aged 18–49 years and 565 older males aged 50–74y years using data envelopment analysis of 8-day dietary record data.

†P values by the Student's t-test. P<0.05 was considered significant.

‡Dietary components to increase

§Dietary components to decrease

# Supplemental Table S5. Comparison of food and nutrient intake between females with efficient diets and inefficient diets (per 2000 kcal).*

|  | Younger | | | | | |  | Older | | | | | |
| --- | --- | --- | --- | --- | --- | --- | --- | --- | --- | --- | --- | --- | --- |
|  | Efficient diets (n=79) | |  | Inefficient diets (n=655) | | P-value† |  | Efficient diets (n=134) | |  | Inefficient diets (n=471) | | P-value† |
|  | Mean | SD |  | Mean | SD |  |  | Mean | SD |  | Mean | SD |  |
| Food intake |  |  |  |  |  |  |  |  |  |  |  |  |  |
| Refined grain | 359.1 | 150.5 |  | 424.9 | 101.7 | <0.0001 |  | 318.7 | 111.9 |  | 377.1 | 87.1 | <0.0001 |
| Whole grain‡ | 34.5 | 66.0 |  | 14.4 | 30.2 | <0.0001 |  | 41.3 | 73.6 |  | 16.4 | 25.6 | <0.0001 |
| Potatoes | 45.3 | 34.6 |  | 39.0 | 23.2 | 0.031 |  | 43.5 | 26.1 |  | 44.2 | 25.1 | 0.77 |
| Total vegetables (excluding pickled vegetables) ‡ | 314.9 | 180.3 |  | 261.4 | 93.5 | <0.0001 |  | 387.2 | 147.7 |  | 325.3 | 96.8 | <0.0001 |
| Green and yellow vegetables | 110.1 | 66.4 |  | 79.5 | 39.5 | <0.0001 |  | 148.7 | 73.9 |  | 109.8 | 48.1 | <0.0001 |
| Other vegetables | 176.6 | 114.5 |  | 158.7 | 62.2 | 0.031 |  | 203.2 | 88.9 |  | 185.9 | 60.6 | 0.009 |
| Mushrooms | 5.7 | 7.4 |  | 5.4 | 6.6 | 0.76 |  | 9.3 | 10.8 |  | 11.3 | 13.0 | 0.12 |
| Seaweed | 17.8 | 24.0 |  | 13.6 | 12.0 | 0.012 |  | 19.6 | 17.9 |  | 17.5 | 13.8 | 0.15 |
| Pickled vegetables | 10.4 | 16.7 |  | 9.6 | 11.0 | 0.54 |  | 15.7 | 20.5 |  | 12.0 | 12.8 | 0.012 |
| Legumes (soy, soy products, other beans and peas) ‡ | 96.0 | 101.5 |  | 53.3 | 44.5 | <0.0001 |  | 102.8 | 82.6 |  | 65.6 | 43.8 | <0.0001 |
| Nuts and seeds‡ | 4.6 | 8.1 |  | 1.8 | 2.4 | <0.0001 |  | 5.7 | 8.2 |  | 2.7 | 3.4 | <0.0001 |
| Oily fish and lean fish‡ | 31.8 | 29.8 |  | 24.0 | 19.6 | 0.002 |  | 48.1 | 33.3 |  | 36.7 | 24.9 | <0.0001 |
| Other fish, seafoods, and processed seafood | 21.9 | 17.3 |  | 23.5 | 16.7 | 0.42 |  | 33.6 | 22.8 |  | 33.2 | 20.5 | 0.83 |
| Red meat and processed meat products§ | 47.4 | 32.0 |  | 67.8 | 28.2 | <0.0001 |  | 48.6 | 30.6 |  | 63.4 | 24.5 | <0.0001 |
| Chicken | 40.1 | 51.1 |  | 37.7 | 24.4 | 0.50 |  | 22.5 | 23.3 |  | 25.2 | 19.1 | 0.17 |
| Eggs | 44.8 | 27.3 |  | 44.2 | 22.8 | 0.82 |  | 42.5 | 20.6 |  | 46.3 | 20.8 | 0.064 |
| Dairy foods‡ | 151.6 | 144.7 |  | 99.7 | 76.0 | <0.0001 |  | 184.4 | 131.0 |  | 130.7 | 86.5 | <0.0001 |
| Fruits‡ | 97.3 | 102.7 |  | 50.3 | 48.4 | <0.0001 |  | 162.9 | 103.2 |  | 110.4 | 68.5 | <0.0001 |
| Fruit and vegetable juice | 34.0 | 62.2 |  | 24.9 | 44.8 | 0.11 |  | 28.8 | 54.5 |  | 25.6 | 51.6 | 0.53 |
| Alcoholic beverages§ | 41.6 | 171.4 |  | 75.7 | 201.9 | 0.15 |  | 43.3 | 129.0 |  | 79.0 | 175.0 | 0.028 |
| Sweetened beverages§ | 39.7 | 76.4 |  | 65.4 | 100.8 | 0.029 |  | 25.9 | 50.3 |  | 40.6 | 79.6 | 0.042 |
| Tea, coffee and waters | 1104.3 | 703.7 |  | 914.7 | 494.4 | 0.002 |  | 1021.7 | 535.0 |  | 933.5 | 408.3 | 0.041 |
| Fats and oils | 12.2 | 5.2 |  | 12.9 | 4.8 | 0.27 |  | 11.5 | 5.9 |  | 13.6 | 5.1 | <0.0001 |
| Sugar and confectionery | 64.8 | 47.1 |  | 55.5 | 33.7 | 0.028 |  | 56.3 | 35.2 |  | 52.2 | 30.9 | 0.19 |
| Seasonings | 91.0 | 51.6 |  | 113.2 | 64.5 | 0.003 |  | 116.8 | 72.3 |  | 125.0 | 67.0 | 0.22 |
| Nutrient intake |  |  |  |  |  |  |  |  |  |  |  |  |  |
| Protein (g) | 76.3 | 17.6 |  | 72.3 | 8.5 | 0.0006 |  | 80.9 | 11.6 |  | 76.8 | 8.6 | <0.0001 |
| Total fat (g) | 67.1 | 12.3 |  | 68.0 | 9.6 | 0.45 |  | 66.7 | 10.3 |  | 67.3 | 9.4 | 0.51 |
| Saturated fat (g) | 19.8 | 5.1 |  | 20.5 | 4.0 | 0.12 |  | 19.1 | 4.0 |  | 19.6 | 3.7 | 0.22 |
| Carbohydrates (g) | 263 | 40 |  | 260 | 27 | 0.32 |  | 262 | 27 |  | 259 | 28 | 0.17 |
| Total fibre (g) | 15.8 | 5.7 |  | 12.7 | 2.7 | <0.0001 |  | 18.6 | 5.0 |  | 15.3 | 3.4 | <0.0001 |
| Vitamin A (μgRAE) | 688 | 544 |  | 539 | 349 | 0.0009 |  | 801 | 538 |  | 657 | 405 | 0.0008 |
| Vitamin D (μg) | 8.3 | 5.2 |  | 6.8 | 3.6 | 0.001 |  | 10.3 | 5.1 |  | 9.0 | 4.4 | 0.003 |
| Vitamin E (mg) | 10.7 | 3.2 |  | 9.2 | 1.8 | <0.0001 |  | 11.5 | 2.5 |  | 10.2 | 1.9 | <0.0001 |
| Vitamin K (μg) | 306.0 | 163.0 |  | 228.3 | 92.7 | <0.0001 |  | 336.3 | 161.5 |  | 274.3 | 108.7 | <0.0001 |
| Thiamine (mg) | 1.06 | 0.27 |  | 1.04 | 0.19 | 0.58 |  | 1.15 | 0.24 |  | 1.10 | 0.19 | 0.011 |
| Riboflavin (mg) | 1.4 | 0.4 |  | 1.2 | 0.2 | <0.0001 |  | 1.6 | 0.3 |  | 1.4 | 0.2 | <0.0001 |
| Niacin (mg) | 18.8 | 8.0 |  | 17.3 | 3.7 | 0.004 |  | 20.6 | 5.3 |  | 19.0 | 3.6 | <0.0001 |
| Vitamin B_6_ (mg) | 1.5 | 0.6 |  | 1.2 | 0.2 | <0.0001 |  | 1.6 | 0.4 |  | 1.4 | 0.3 | <0.0001 |
| Vitamin B_12_ (μg) | 5.7 | 3.6 |  | 5.2 | 3.0 | 0.14 |  | 8.0 | 4.1 |  | 6.9 | 3.3 | 0.002 |
| Folate (μg) | 409 | 168 |  | 326 | 110 | <0.0001 |  | 482 | 140 |  | 403 | 105 | <0.0001 |
| Pantothenic acid (mg) | 6.6 | 1.7 |  | 5.7 | 0.9 | <0.0001 |  | 7.0 | 1.2 |  | 6.2 | 0.9 | <0.0001 |
| Vitamin C (mg) | 131 | 59 |  | 100 | 38 | <0.0001 |  | 168 | 59 |  | 136 | 46 | <0.0001 |
| Sodium (g salt)§ | 8.8 | 2.2 |  | 10.2 | 1.7 | <0.0001 |  | 9.9 | 2.0 |  | 10.9 | 1.8 | <0.0001 |
| Potassium (mg) | 2947 | 885 |  | 2446 | 487 | <0.0001 |  | 3417 | 701 |  | 2925 | 542 | <0.0001 |
| Calcium (mg) | 615 | 210 |  | 495 | 133 | <0.0001 |  | 714 | 195 |  | 584 | 137 | <0.0001 |
| Magnesium (mg) | 301 | 91 |  | 252 | 48 | <0.0001 |  | 349 | 75 |  | 296 | 49 | <0.0001 |
| Iron (mg) | 9.0 | 2.9 |  | 7.7 | 1.5 | <0.0001 |  | 10.1 | 2.2 |  | 8.8 | 1.6 | <0.0001 |
| Zinc (mg) | 8.7 | 1.4 |  | 8.6 | 1.1 | 0.41 |  | 9.3 | 1.5 |  | 8.8 | 1.1 | <0.0001 |
| Copper (mg) | 1.3 | 0.3 |  | 1.1 | 0.2 | <0.0001 |  | 1.4 | 0.2 |  | 1.2 | 0.2 | <0.0001 |
| Alcohol (g) | 3.6 | 13.2 |  | 4.9 | 12.1 | 0.38 |  | 3.3 | 7.4 |  | 5.2 | 10.7 | 0.063 |
| Added sugar (g) | 35.0 | 20.9 |  | 32.7 | 15.0 | 0.20 |  | 30.3 | 13.8 |  | 29.7 | 12.5 | 0.66 |

RAE, Retinol Activity Equivalents.

*Efficient diets were identified separately for 734 younger females aged 18–49 years and 605 older females aged 50–74 years by data envelopment analysis of 8-day dietary record data.

†P values by Student's t-test for continuous variables and chi-squared test for categorical variables. P<0.05 was considered significant.

‡Dietary components to increase

§Dietary components to decrease

# Supplemental Table S6. Comparison of basic characteristics between participants with and without feasible solutions (males).*

|  | Younger males (18–49 y) | | | | | |  | Older males (50–74 y) | | | | | |
| --- | --- | --- | --- | --- | --- | --- | --- | --- | --- | --- | --- | --- | --- |
|  | Without a feasible solution (n=8) | |  | With a feasible solution (n=572) | | P-value† |  | Without a feasible solution(n=24) | |  | With a feasible solution (n=410) | | P-value† |
|  | Mean or N | SD or (%) | | Mean or N | SD or (%) |  |  | Mean or N | SD or (%) | | Mean or N | SD or (%) |  |
| Age (y) | 34.6 | 6.6 |  | 33.4 | 8.9 | 0.70 |  | 58.8 | 7.3 |  | 61.9 | 7.1 | 0.04 |
| Body height (cm) | 172.1 | 5.8 |  | 171.2 | 6.0 | 0.71 |  | 169.3 | 5.7 |  | 168.3 | 5.7 | 0.41 |
| Body weight (kg) | 70.1 | 10.1 |  | 68.9 | 12.7 | 0.79 |  | 67.0 | 8.5 |  | 67.7 | 8.8 | 0.71 |
| Body mass index (kg/m^2^) | 23.6 | 2.5 |  | 23.5 | 3.9 | 0.90 |  | 23.4 | 2.5 |  | 23.9 | 2.8 | 0.36 |
| Residential area, N (%) |  |  |  |  |  | 0.23 |  |  |  |  |  |  | 0.43 |
| Hokkaido and Tohoku | 2 | (25) |  | 62 | (11) |  |  | 2 | (8) |  | 53 | (13) |  |
| Kanto | 4 | (50) |  | 173 | (30) |  |  | 8 | (33) |  | 125 | (30) |  |
| Hokuriku and Tokai | 1 | (13) |  | 120 | (21) |  |  | 7 | (29) |  | 83 | (20) |  |
| Kinki, Chugoku and Shikoku | 0 | (0) |  | 140 | (24) |  |  | 3 | (13) |  | 105 | (26) |  |
| Kyusyu and Okinawa | 1 | (13) |  | 77 | (13) |  |  | 4 | (17) |  | 44 | (11) |  |
| Annual household income, N (%) |  |  |  |  |  | 0.09 |  |  |  |  |  |  | 0.70 |
| <5 million Japanese yen | 7 | (88) |  | 238 | (42) |  |  | 12 | (50) |  | 179 | (44) |  |
| ≥5 to <8 million Japanese yen | 1 | (13) |  | 189 | (33) |  |  | 8 | (33) |  | 125 | (30) |  |
| ≥8 million Japanese yen | 0 | (0) |  | 134 | (23) |  |  | 4 | (17) |  | 104 | (25) |  |
| Missing | 0 | (0) |  | 11 | (2) |  |  | 0 | (0) |  | 2 | (0) |  |
| Educational level, N (%) |  |  |  |  |  | 0.50 |  |  |  |  |  |  | 0.26 |
| Junior high or high school | 1 | (13) |  | 177 | (31) |  |  | 16 | (67) |  | 185 | (45) |  |
| Junior college or technical school | 2 | (25) |  | 151 | (26) |  |  | 2 | (8) |  | 50 | (12) |  |
| University or higher | 5 | (63) |  | 237 | (41) |  |  | 6 | (25) |  | 172 | (42) |  |
| Other | 0 | (0) |  | 2 | (0) |  |  | 0 | (0) |  | 3 | (1) |  |
| Missing | 0 | (0) |  | 5 | (1) |  |  |  | (0) |  |  | (0) |  |
| Employment status, N (%) |  |  |  |  |  | 1.00 |  |  |  |  |  |  | 0.91 |
| Unemployed | 0 | (0) |  | 5 | (1) |  |  | 6 | (25) |  | 90 | (22) |  |
| Students | 0 | (0) |  | 48 | (8) |  |  | 0 | (0) |  | 0 | (0) |  |
| Part-time job | 0 | (0) |  | 10 | (2) |  |  | 3 | (13) |  | 64 | (16) |  |
| Full-time job | 8 | (100) |  | 505 | (88) |  |  | 15 | (63) |  | 256 | (62) |  |
| Missing | 0 | (0) |  | 4 | (1) |  |  |  | (0) |  |  | (0) |  |
| Smoking status, N (%) |  |  |  |  |  | 0.49 |  |  |  |  |  |  | 0.60 |
| Current smoker | 2 | (25) |  | 175 | (31) |  |  | 8 | (33) |  | 103 | (25) |  |
| Past smoker | 3 | (38) |  | 110 | (19) |  |  | 10 | (42) |  | 208 | (51) |  |
| Non-smoker | 3 | (38) |  | 283 | (49) |  |  | 6 | (25) |  | 99 | (24) |  |
| Missing | 0 | (0) |  | 4 | (1) |  |  | 0 | (0) |  | 0 | (0) |  |
| Energy intake (kcal/day) | 1909 | 420 |  | 2240 | 516 | 0.07 |  | 2259 | 334 |  | 2313 | 437 | 0.55 |
| NRF9.3 score | 506 | 108 |  | 574 | 109 | 0.08 |  | 660 | 91 |  | 679 | 82 | 0.27 |
| Greenhouse gas emissions (kgCO_2_-eq/day) | 3.13 | 0.47 |  | 3.61 | 0.71 | 0.06 |  | 3.14 | 0.50 |  | 3.72 | 0.58 | 0.47 |
| Eutrophication (gPO_4_^3-^-eq/day) | 89 | 12 |  | 98 | 16 | 0.13 |  | 89 | 11 |  | 92 | 13 | 0.16 |
| Acidification (gSO_2_-eq/day) | 2.43 | 0.64 |  | 3.07 | 1.18 | 0.13 |  | 2.42 | 0.69 |  | 3.42 | 1.03 | <.0001 |
| Land use change (m^2^ X 10^-3^/day) | 45.3 | 6.1 |  | 49.4 | 7.6 | 0.13 |  | 46.4 | 6.4 |  | 48.9 | 6.4 | 0.06 |
| Water use (L/day) | 2229 | 364 |  | 2200 | 708 | 0.91 |  | 2081 | 512 |  | 2316 | 719 | 0.12 |
| Monetary diet cost (Japanese yen/day) | 905 | 153 |  | 1193 | 202 | <.0001 |  | 1112 | 166 |  | 1371 | 210 | <.0001 |

*Participants were classified according to whether feasible solutions were obtained for the three optimised diet models.

†P values by Student's t-test for continuous variables and chi-squared test for categorical variables. P<0.05 was considered significant.

# Supplemental Table S7. Comparison of basic characteristics between participants with and without feasible solutions (females).*

|  | Younger females (18–49 y) | | | | | |  | Older females (50–74 y) | | | | | |
| --- | --- | --- | --- | --- | --- | --- | --- | --- | --- | --- | --- | --- | --- |
|  | Without a feasible solution (n=39) | |  | With a feasible solution (n=616) | | P-value† |  | Without a feasible solution (n=31) | |  | With a feasible solution (n=440) | | P-value† |
|  | Mean or N | SD or (%) |  | Mean or N | SD or (%) |  |  | Mean or N | SD or (%) |  | Mean or N | SD or (%) |  |
| Age (y) | 30.1 | 9.0 |  | 33.4 | 9.4 | 0.03 |  | 62.6 | 6.8 |  | 61.4 | 7.4 | 0.38 |
| Body height (cm) | 157.9 | 5.8 |  | 158.2 | 5.4 | 0.70 |  | 154.6 | 6.2 |  | 154.9 | 5.6 | 0.79 |
| Body weight (kg) | 52.9 | 9.2 |  | 54.5 | 9.2 | 0.29 |  | 55.6 | 7.7 |  | 55.3 | 9.0 | 0.87 |
| Body mass index (kg/m2) | 21.2 | 3.5 |  | 21.8 | 3.5 | 0.34 |  | 23.3 | 3.1 |  | 23.0 | 3.5 | 0.73 |
| Residential area, N (%) |  |  |  |  |  | 0.51 |  |  |  |  |  |  | 0.49 |
| Hokkaido and Tohoku | 2 | (5) |  | 76 | (12) |  |  | 4 | (13) |  | 46 | (10) |  |
| Kanto | 11 | (28) |  | 187 | (30) |  |  | 7 | (23) |  | 149 | (34) |  |
| Hokuriku and Tokai | 7 | (18) |  | 126 | (20) |  |  | 9 | (29) |  | 79 | (18) |  |
| Kinki, Chugoku and Shikoku | 14 | (36) |  | 156 | (25) |  |  | 8 | (26) |  | 122 | (28) |  |
| Kyusyu and Okinawa | 5 | (13) |  | 71 | (12) |  |  | 3 | (10) |  | 44 | (10) |  |
| Annual household income, N (%) |  |  |  |  |  | 0.17 |  |  |  |  |  |  | 0.02 |
| <5 million Japanese yen | 23 | (59) |  | 263 | (43) |  |  | 24 | (77) |  | 218 | (50) |  |
| ≥5 to <8 million Japanese yen | 6 | (15) |  | 174 | (28) |  |  | 5 | (16) |  | 115 | (26) |  |
| ≥8 million Japanese yen | 9 | (23) |  | 165 | (27) |  |  | 2 | (6) |  | 106 | (24) |  |
| Missing | 1 | (3) |  | 14 | (2) |  |  | 0 | (0) |  | 1 | (0) |  |
| Educational level, N (%) |  |  |  |  |  | 0.36 |  |  |  |  |  |  | 0.34 |
| Junior high or high school | 8 | (21) |  | 196 | (32) |  |  | 16 | (52) |  | 209 | (48) |  |
| Junior college or technical school | 21 | (54) |  | 252 | (41) |  |  | 10 | (32) |  | 178 | (40) |  |
| University or higher | 10 | (26) |  | 165 | (27) |  |  | 4 | (13) |  | 49 | (11) |  |
| Other | 0 | (0) |  | 3 | (0) |  |  | 1 | (3) |  | 2 | (0) |  |
| Missing | 0 | (0) |  | 0 | (0) |  |  | 0 | (0) |  | 2 | (0) |  |
| Employment status, N (%) |  |  |  |  |  | 0.88 |  |  |  |  |  |  | 0.14 |
| Unemployed | 1 | (3) |  | 26 | (4) |  |  | 14 | (45) |  | 123 | (28) |  |
| Students | 5 | (13) |  | 59 | (10) |  |  | 0 | (0) |  | 0 | (0) |  |
| Part-time job | 3 | (8) |  | 62 | (10) |  |  | 5 | (16) |  | 109 | (25) |  |
| Full-time job | 30 | (77) |  | 469 | (76) |  |  | 12 | (39) |  | 208 | (47) |  |
| Missing | 0 | (0) |  | 0 | (0) |  |  | 0 | (0) |  | 0 | (0) |  |
| Smoking status, N (%) |  |  |  |  |  |  |  |  |  |  |  |  | 0.21 |
| Current smoker | 2 | (5) |  | 75 | (12) | 0.36 |  | 3 | (10) |  | 22 | (5) |  |
| Past smoker | 3 | (8) |  | 67 | (11) |  |  | 5 | (16) |  | 49 | (11) |  |
| Non-smoker | 34 | (87) |  | 474 | (77) |  |  | 23 | (74) |  | 369 | (84) |  |
| Energy intake (kcal) | 1664 | 289 |  | 1771 | 342 | 0.06 |  | 1790 | 292 |  | 1920 | 296 | 0.02 |
| NRF9.3 score | 599 | 102 |  | 587 | 109 | 0.49 |  | 658 | 114 |  | 681 | 79 | 0.12 |
| Greenhouse gas emissions (kgCO_2_-eq/day) | 2.38 | 0.52 |  | 2.89 | 0.48 | <.0001 |  | 2.61 | 0.42 |  | 3.14 | 0.49 | <.0001 |
| Eutrophication (gPO_4_^3-^-eq/day) | 75.7 | 13.0 |  | 79.9 | 10.3 | 0.015 |  | 73.0 | 11.5 |  | 78.2 | 10.3 | 0.007 |
| Acidification (gSO_2_-eq/day) | 1.67 | 0.48 |  | 2.33 | 0.73 | <.0001 |  | 2.00 | 0.48 |  | 2.63 | 0.71 | <.0001 |
| Land use change (m^2^/day) | 38.7 | 6.3 |  | 41.0 | 5.3 | 0.011 |  | 38.7 | 6.2 |  | 41.6 | 5.0 | 0.002 |
| Water use (L/day) | 1507 | 316 |  | 1697 | 515 | 0.02 |  | 1674 | 375 |  | 1943 | 535 | 0.006 |
| Monetary diet cost (Japanese yen/day) | 813 | 125 |  | 1015 | 159 | <.0001 |  | 958 | 166 |  | 1161 | 190 | <.0001 |

*Participants were classified according to whether feasible solutions were obtained for the three optimised diet models.

†P values by Student's t-test for continuous variables and chi-squared test for categorical variables. P<0.05 was considered significant.

# Supplemental Table S8. Comparison of food intake between observed and optimised diets among males with inefficient diets (per 2500 kcal)*

|  | Younger (aged 18–49 years; n=572) | | | | | | | | | | | | | |  |  | Older (aged 50–74 years; n=410) | | | | | | | | | | | | | |  |
| --- | --- | --- | --- | --- | --- | --- | --- | --- | --- | --- | --- | --- | --- | --- | --- | --- | --- | --- | --- | --- | --- | --- | --- | --- | --- | --- | --- | --- | --- | --- | --- |
|  | Observed diets | |  |  | MaxNRF† | |  |  | MinGHGE‡ | |  |  | OPT2G§ | |  |  | Observed diets | |  |  | MaxNRF† | |  |  | MinGHGE‡ | |  |  | OPT2G§ | |  |
|  | Mean | SD |  |  | Mean | SD |  |  | Mean | SD |  |  | Mean | SD |  |  | Mean | SD |  |  | Mean | SD |  |  | Mean | SD |  |  | Mean | SD |  |
| Refined grain | 601 | 139 | a |  | **535** | **128** | b |  | **804** | **155** | c |  | 620 | 208 | a |  | 522 | 129 | a |  | 565 | 106 | b |  | 491 | 121 | c |  | **582** | **95** | b |
| Whole grain | 14.2 | 29.2 | a |  | **107** | **83.9** | b |  | **37.6** | **34.6** | c |  | **127** | **117** | d |  | 17.5 | 32.9 | a |  | **32.0** | **37.4** | b |  | **47.0** | **34.7** | c |  | **31.1** | **31.9** | b |
| Potatoes | 40.2 | 26.8 | a |  | **47.9** | **12.2** | b |  | **29.7** | **18.2** | c |  | **48.6** | **15.9** | b |  | 46.6 | 26.7 |  |  | 47.6 | 9.9 |  |  | 46.8 | 15.6 |  |  | 46.9 | 10.8 |  |
| Total vegetables | 250 | 98 | a |  | **323** | **76** | b |  | **316** | **63.7** | b |  | **326** | **70.4** | b |  | 335 | 114 | a |  | **413** | **75** | b |  | 364 | 92 | c |  | **435** | **93** | d |
| Pickled vegetables | 7.8 | 8.6 | a |  | 8.7 | 7.1 | a |  | **13.8** | **8.4** | b |  | 7.8 | 7.0 | a |  | 14.4 | 15.5 | a |  | **10.2** | **8.7** | c |  | 15.2 | 8.2 | a |  | 15.4 | 7.9 | a |
| Legumes | 45.3 | 35.4 | a |  | **78.4** | **29.5** | b |  | **61.3** | **31.7** | c |  | **90.7** | **30.0** | d |  | 73.5 | 50.8 | a |  | **131** | **37.6** | b |  | **127** | **63.7** | b |  | **118** | **38.8** | d |
| Nuts and seeds | 1.8 | 2.7 | a |  | **3.2** | **3.5** | b |  | **7.4** | **9.3** | c |  | **5.5** | **7.6** | d |  | 2.5 | 3.9 | a |  | **3.2** | **3.7** | ab |  | **6.1** | **4.4** | c |  | **3.4** | **3.6** | b |
| Fish | 52.1 | 31.8 | a |  | **65.9** | **24.9** | b |  | **65.5** | **22.4** | b |  | **75.8** | **28.5** | c |  | 87.1 | 40.0 | a |  | **122** | **25.0** | b |  | 89.5 | 22.7 | a |  | **100** | **25.3** | c |
| Red meat and processed meat products | 96.3 | 45.1 | a |  | **60.5** | **21.1** | b |  | **38.8** | **13.4** | c |  | **35.7** | **15.2** | c |  | 81.1 | 32.4 | a |  | **60.7** | **17.3** | b |  | **39.3** | **16.3** | c |  | **53.1** | **16.5** | d |
| Chicken | 51.0 | 38.2 | a |  | 49.8 | 22.9 | a |  | **31.5** | **15.7** | b |  | **25.4** | **19.4** | c |  | 35.6 | 28.3 | a |  | **27.3** | **8.5** | b |  | **24.1** | **8.8** | c |  | **25.2** | **9.8** | bc |
| Eggs | 48.1 | 25.4 | a |  | 43.8 | 17.8 | b |  | **39.9** | **16.1** | c |  | **31.6** | **16.9** | d |  | 53.8 | 25.2 | a |  | **44.5** | **17.7** | b |  | 51.8 | 11.6 | a |  | **63.0** | **19.9** | c |
| Dairy foods | 79.4 | 81.8 | a |  | **156** | **102** | b |  | **99.4** | **79.3** | c |  | **107** | **82.3** | c |  | 113 | 96.4 | a |  | **161** | **76.5** | b |  | **151** | **79.3** | bc |  | **143** | **83.4** | c |
| Fruits | 28.9 | 37.6 | a |  | **92.3** | **52.5** | b |  | **56.5** | **41.7** | c |  | **104** | **74.9** | d |  | 82.0 | 70.3 | a |  | **114** | **70.3** | b |  | **127** | **64.8** | c |  | **132** | **56.8** | c |
| Fruit and vegetable juice | 24.3 | 52.7 | a |  | **9.5** | **16.7** | b |  | 31.2 | 41.2 | c |  | 25.6 | 35.9 | ac |  | 22.8 | 47.0 | ab |  | 24.2 | 29.1 | a |  | **17.4** | **14.1** | b |  | **17.6** | **14.9** | b |
| Alcoholic beverages | 180 | 334 | a |  | **29.9** | **47.6** | b |  | **29.9** | **51.7** | b |  | **41.9** | **49.5** | b |  | 283 | 304 | a |  | **144** | **153.7** | b |  | **155** | **137.4** | b |  | **101** | **94.5** | c |
| Sweetened beverages | 137 | 183 | a |  | **77.1** | **84.3** | b |  | **25.8** | **47.6** | c |  | **37.0** | **48.0** | c |  | 62.1 | 111.7 | a |  | **10.0** | **21.1** | b |  | **11.4** | **20.2** | b |  | **11.5** | **21.6** | b |
| Tea, coffee and water | 1043 | 541 | a |  | **1246** | **394** | b |  | **839** | **291** | c |  | 1020 | 454 | a |  | 954 | 430 | a |  | **1074** | **286** | b |  | **840** | **248** | c |  | 949 | 291 | b |
| Fats and oils | 14.9 | 5.9 | a |  | 14.7 | 2.4 | a |  | **9.7** | **3.5** | b |  | 13.8 | 2.5 | c |  | 14.7 | 5.9 | a |  | **11.4** | **3.7** | b |  | 14.1 | 3.1 | ac |  | 13.4 | 3.6 | c |
| Sugar and confectionery | 44.0 | 36.3 | a |  | 40.0 | 16.6 | b |  | **18.3** | **18.9** | c |  | **30.0** | **18.3** | d |  | 45.4 | 35.1 | a |  | **28.7** | **11.5** | b |  | **37.9** | **15.6** | c |  | **32.6** | **12.7** | d |
| Seasonings | 143 | 78.7 | a |  | **116** | **38.3** | b |  | **112** | **44.7** | b |  | **111** | **55.7** | b |  | 143 | 72.9 | a |  | **179** | **50.2** | b |  | **122** | **55.8** | c |  | **203** | **63.9** | d |

Values changed by >10% from the observed value and statistically significant (P<0.05 by repeated ANOVA with Bonferroni adjustment) are written in bold.

Values with different letters within a row (a,b,c,d) are significantly different from the values in observed diets or other optimised diets by repeated-measures ANOVA (two-sided P< 0·05 with Bonferroni adjustment). For example in total refined grain (younger men), there is no significant difference between observed diet and OPT2G (both with "a"), but MaxNRF (b) and MinGHGE (c) are significantly different from each other and from observed diet and OPT2G.

*Those for whom a feasible solution could not be obtained were excluded from the analysis (8 young and 24 older male participants).

†Optimised diets targeted to maximise the Nutrient Rich Food index 9.3.

‡Optimised diets targeted to minimise diet-related greenhouse gas emissions.

§Optimised diets target to achieve two goals (i.e., maximise the Nutrient Rich Food index 9.3 and minimise diet-related greenhouse gas emissions) with equal weight.

# Supplemental Table S9. Comparison of food intake between observed and optimised diets among females with inefficient diets (per 2000 kcal)*

|  | Younger (aged 18–49 years; n=616) | | | | | | | | | | | | | |  |  | Older (aged 50–74 years; n=440) | | | | | | | | | | | | | |  |
| --- | --- | --- | --- | --- | --- | --- | --- | --- | --- | --- | --- | --- | --- | --- | --- | --- | --- | --- | --- | --- | --- | --- | --- | --- | --- | --- | --- | --- | --- | --- | --- |
|  | Observed diets | |  |  | MaxNRF† | |  |  | MinGHGE‡ | |  |  | OPT2G§ | |  |  | Observed diets | |  |  | MaxNRF† | |  |  | MinGHGE‡ | |  |  | OPT2G§ | |  |
|  | Mean | SD |  |  | Mean | SD |  |  | Mean | SD |  |  | Mean | SD |  |  | Mean | SD |  |  | Mean | SD |  |  | Mean | SD |  |  | Mean | SD |  |
| **Females (per 2000 kcal)** |  |  |  |  |  |  |  |  |  |  |  |  |  |  |  |  |  |  |  |  |  |  |  |  |  |  |  |  |  |  |  |
| Refined grain | 422 | 99.2 | a |  | **371** | **90.5** | b |  | 450 | 90.6 | c |  | 421 | 91.1 | a |  | 375 | 85.9 | a |  | **336** | **67.8** | b |  | 383 | 73.2 | ac |  | 392 | 85.7 | c |
| Whole grain | 14.2 | 29.7 | a |  | **42.0** | **38.6** | b |  | **53.3** | **51.5** | c |  | **41.3** | **40.5** | b |  | 16.1 | 25.1 | a |  | **52.2** | **49.5** | b |  | **31.5** | **22.5** | c |  | **20.5** | **24.4** | a |
| Potatoes | 38.9 | 23.1 | a |  | **69.5** | **23.1** | b |  | 41.1 | 19.6 | a |  | **79.4** | **23.8** | c |  | 44.3 | 25.1 | a |  | **35.1** | **12.0** | b |  | **62.2** | **19.3** | c |  | **39.6** | **12.8** | d |
| Total vegetables | 261 | 91.3 | a |  | **293** | **79.4** | b |  | 276 | 83.0 | c |  | 285 | 79.4 | b,c |  | 325 | 94.8 | a |  | **370** | **67.5** | b |  | 343 | 80.1 | c |  | 349 | 76.0 | c |
| Pickled vegetables | 5.5 | 6.7 | a |  | **3.4** | **2.4** | b |  | **6.5** | **4.6** | c |  | **3.2** | **2.5** | b |  | 11.6 | 13.1 | a |  | **9.1** | **6.6** | b |  | **8.0** | **4.6** | b |  | **9.0** | **4.9** | b |
| Legumes | 52.3 | 43.1 | a |  | **93.5** | **35.9** | b |  | **97.7** | **55.8** | b |  | **94.3** | **36.8** | b |  | 65.5 | 43.6 | a |  | **147.0** | **52.9** | b |  | **104** | **36.8** | c |  | **108** | **39.4** | c |
| Nuts and seeds | 1.8 | 2.4 | a |  | **4.3** | **3.9** | b |  | **6.4** | **4.1** | c |  | **3.8** | **3.3** | b |  | 2.7 | 3.4 | a |  | **5.3** | **5.3** | b |  | **5.3** | **3.5** | b |  | **4.8** | **3.5** | b |
| Fish | 48.5 | 28.0 | a |  | **63.2** | **22.5** | b |  | **56.8** | **20.3** | c |  | **65.1** | **22.3** | b |  | 70.7 | 34.5 | a |  | 65.3 | 22.5 | b |  | **63.6** | **27.0** | bc |  | **59.8** | **21.0** | c |
| Red meat and processed meat products | 68.3 | 27.6 | a |  | **43.4** | **15.7** | b |  | **26.1** | **12.1** | c |  | **30.4** | **7.9** | d |  | 63.3 | 24.0 | a |  | **42.4** | **13.5** | b |  | **35.8** | **9.8** | c |  | **38.9** | **9.7** | d |
| Chicken | 37.9 | 24.3 | a |  | 41.1 | 12.9 | b,c |  | 40.8 | 21.4 | b |  | **43.8** | **11.4** | c |  | 25.3 | 19.1 | a |  | **21.9** | **14.2** | b |  | **35.3** | **16.3** | c |  | **28.0** | **14.5** | a |
| Eggs | 43.7 | 21.7 | a |  | 47.7 | 10.6 | b |  | **35.7** | **8.1** | c |  | 42.4 | 8.6 | a |  | 45.8 | 21.0 | a |  | **37.5** | **12.8** | b |  | 42.6 | 9.1 | c |  | **38.7** | **12.0** | b |
| Dairy foods | 98.9 | 73.6 | a |  | **209** | **80.5** | b |  | **164** | **59.0** | c |  | **158** | **53.0** | c |  | 130 | 85.0 | a |  | **156** | **68.6** | b |  | **228** | **66.1** | c |  | **160** | **63.8** | b |
| Fruits | 50.7 | 48.7 | a |  | **102** | **42.6** | b |  | **81.2** | **51.7** | c |  | **117** | **39.1** | d |  | 112 | 66.6 | a |  | **201** | **58.6** | b |  | **136** | **53.2** | c |  | **218** | **57.3** | d |
| Fruit and vegetable juice | 25.1 | 45.2 | a |  | **32.8** | **31.2** | b |  | **6.3** | **16.8** | c |  | **29.6** | **30.0** | a,b |  | 26.3 | 53.1 | a |  | **16.5** | **17.0** | b |  | 26.2 | 24.2 | a |  | **14.8** | **14.4** | b |
| Alcoholic beverages | 79.5 | 207.4 | a |  | **8.5** | **26.2** | b |  | **2.2** | **13.4** | b |  | **3.3** | **9.5** | b |  | 83.3 | 179.4 | a |  | **3.7** | **5.9** | b |  | **9.0** | **17.4** | b |  | **3.3** | **7.1** | b |
| Sweetened beverages | 67.4 | 102.5 | a |  | **33.2** | **34.8** | b |  | **7.9** | **12.5** | c |  | **18.4** | **28.5** | d |  | 40.8 | 80.0 | a |  | **4.5** | **9.7** | b |  | **4.7** | **8.3** | b |  | **4.8** | **9.9** | b |
| Tea, coffee and waters | 917 | 490 | a |  | **1161** | **360** | b |  | **803** | **205** | c |  | **1317** | **368** | d |  | 951 | 411 | a |  | 923 | 238 | a |  | **691** | **194** | b |  | **835** | **223** | c |
| Fats and oils | 12.8 | 4.9 | a |  | 13.3 | 2.9 | a,b |  | **10.9** | **3.6** | c |  | 13.6 | 3.4 | b |  | 13.5 | 5.0 | a |  | 14.1 | 4.1 | a |  | **10.1** | **2.4** | b |  | **12.0** | **3.4** | c |
| Sugar and confectionery | 56.3 | 34.1 | a |  | **43.2** | **18.7** | b |  | **48.2** | **14.0** | c |  | **37.7** | **18.7** | d |  | 53.0 | 31.0 | a |  | 56.0 | 17.2 | a |  | **34.5** | **15.2** | b |  | 48.5 | 15.0 | c |
| Seasonings | 113 | 65.0 | a |  | 104 | 35.7 | b |  | **92** | **35.4** | c |  | 114 | 32.0 | b |  | 125 | 65.6 | a |  | 122 | 44.3 | a |  | **109** | **36** | b |  | 120 | 49.8 | a |

Values changed by >10% from the observed value and statistically significant (P<0.05 by repeated ANOVA with Bonferroni adjustment) are written in bold.

Values with different letters within a row (a,b,c,d) are significantly different from the values in observed diets or other optimised diets by repeated-measures ANOVA (two-sided P< 0·05 with Bonferroni adjustment). Values sharing the same letter are not significantly different.

*Those for whom a feasible solution could not be obtained were excluded from the analysis (39 younger and 31 older female participants).

†Optimised diets targeted to maximise the Nutrient Rich Food index 9.3.

‡Optimised diets targeted to minimise diet-related greenhouse gas emissions.

§Optimised diets target to achieve two goals (i.e., maximise the Nutrient Rich Food index 9.3 and minimise diet-related greenhouse gas emissions) with equal weight.

# Supplemental Table S10. Comparison of NRF9.3 score, environmental impacts, and monetary cost of observed diets and optimised diets among males with inefficient diets (per 2500 kcal)*

|  | Younger males (aged 18–49 years; n=572) | | | | | | | | | | | | | | |  | Older males (aged 50–74 years; n=410) | | | | | | | | | | | | | | |
| --- | --- | --- | --- | --- | --- | --- | --- | --- | --- | --- | --- | --- | --- | --- | --- | --- | --- | --- | --- | --- | --- | --- | --- | --- | --- | --- | --- | --- | --- | --- | --- |
|  | Observed diets | |  |  | MaxNRF† | |  |  | MinGHGE‡ | |  |  | OPT2G§ | |  |  | Observed diets | |  |  | MaxNRF† | |  |  | MinGHGE‡ | |  |  | OPT2G§ | |  |
|  | Mean | SD |  |  | Mean | SD |  |  | Mean | SD |  |  | Mean | SD |  |  | Mean | SD |  |  | Mean | SD |  |  | Mean | SD |  |  | Mean | SD |  |
| NRF 9.3 score | 574 | 109 | a |  | **736** | **67** | b |  | **649** | **75** | c |  | **743** | **78** | b |  | 679 | 82 | a |  | **786** | **29** | b |  | **767** | **48** | c |  | **778** | **41** | b |
| Greenhouse gas emissions (kgCO_2_-eq) | 3.61 | 0.71 | a |  | 3.32 | 0.42 | b |  | **2.35** | **0.35** | c |  | **2.61** | **0.28** | d |  | 3.72 | 0.58 | a |  | 3.77 | 0.29 | a |  | **2.87** | **0.39** | b |  | **3.18** | **0.30** | c |
| Acidification (gSO_2_-eq) | 3.07 | 1.18 | a |  | **2.52** | **0.39** | b |  | **1.62** | **0.35** | c |  | **1.91** | **0.30** | d |  | 3.42 | 1.03 | a |  | **3.11** | **0.46** | b |  | **2.25** | **0.35** | c |  | **2.51** | **0.33** | d |
| Eutrophication (gPO_4_^3-^-eq) | 97.6 | 16.1 | a |  | 94.9 | 12.8 | b |  | **73.6** | **9.3** | c |  | **75.7** | **9.9** | d |  | 92.4 | 13.0 | a |  | 91.7 | 7.3 | a |  | **82.6** | **7.2** | b |  | **84.1** | **6.3** | b |
| Land use change (m^2^×10^-3^) | 49.4 | 7.6 | a |  | 48.2 | 5.0 | b |  | **37.3** | **5.2** | c |  | **40.1** | **4.6** | d |  | 48.9 | 6.4 | a |  | 47.9 | 4.2 | b |  | **46.1** | **3.6** | c |  | **44.7** | **3.7** | d |
| Water resources consumption (L) | 2200 | 708 | a |  | 1984 | 345 | b |  | **1725** | **189** | c |  | **1661** | **281** | c |  | 2316 | 719 | a |  | 2276 | 262 | a |  | **1949** | **316** | b |  | **2252** | **290** | a |
| Monetary cost (JPN) | 1193 | 202 | a |  | 1135 | 109 | b |  | **928** | **117** | c |  | **1013** | **101** | d |  | 1371 | 210 | a |  | 1356 | 183 | a |  | **1251** | **143** | b |  | **1314** | **179** | c |

NRF, Nutrient Rich Food Index; JPN, Japanese yen.

Values changed by >10% from the observed value and statistically significant (P<0.05 by repeated-measures ANOVA with Bonferroni adjustment) are written in bold.

Values with different letters within a row (a,b,c,d) are significantly different from the values in observed diets or other optimised diets by repeated-measures ANOVA (two-sided P< 0·05 with Bonferroni adjustment). Values sharing the same letter are not significantly different.

*Those for whom a feasible solution could not be obtained were excluded from the analysis (8 younger and 24 older males).

†Optimised diets targeted to maximise the Nutrient Rich Food index 9.3.

‡Optimised diets targeted to minimise diet-related greenhouse gas emissions.

§Optimised diets target to achieve two goals (i.e., maximise the Nutrient Rich Food index 9.3 and minimise diet-related greenhouse gas emissions) with equal weight.

# Supplemental Table S11. Comparison of NRF9.3 score, environmental impacts, and monetary cost of observed diets and optimised diets among females with inefficient diets (per 2000 kcal)*

|  | Younger females (aged 18–49 y; n=616) | | | | | | | | | | | | | | |  | Older females (50–74 y; n=440) | | | | | | | | | | | | | |  |
| --- | --- | --- | --- | --- | --- | --- | --- | --- | --- | --- | --- | --- | --- | --- | --- | --- | --- | --- | --- | --- | --- | --- | --- | --- | --- | --- | --- | --- | --- | --- | --- |
|  | Observed diets | |  |  | MaxNRF† | |  |  | MinGHGE‡ | |  |  | OPT2G§ | |  |  | Observed diets | |  |  | MaxNRF† | |  |  | MinGHGE‡ | |  |  | OPT2G§ | |  |
|  | Mean | SD |  |  | Mean | SD |  |  | Mean | SD |  |  | Mean | SD |  |  | Mean | SD |  |  | Mean | SD |  |  | Mean | SD |  |  | Mean | SD |  |
| NRF 9.3 score | 587 | 109 | a |  | **747** | **62** | b |  | **749** | **67** | b |  | **752** | **61** | b |  | 681 | 79 | a |  | **763** | **35** | b |  | **781** | **38** | c |  | **768** | **37** | b |
| Greenhouse gas emissions (kgCO_2_-eq) | 2.89 | 0.48 | a |  | 2.87 | 0.38 | a |  | **2.18** | **0.20** | b |  | **2.46** | **0.18** | c |  | 3.14 | 0.49 | a |  | 3.14 | 0.30 | a |  | **2.43** | **0.27** | b |  | **2.66** | **0.25** | c |
| Acidification (gSO_2_-eq) | 2.33 | 0.73 | a |  | 2.11 | 0.24 | b |  | **1.60** | **0.24** | c |  | **1.94** | **0.19** | d |  | 2.63 | 0.71 | a |  | 2.52 | 0.31 | b |  | **1.81** | **0.29** | c |  | **2.04** | **0.26** | d |
| Eutrophication (gPO_4_^3-^-eq) | 79.9 | 10.3 | a |  | 84.5 | 12.8 | b |  | **72.6** | **5.9** | c |  | 72.6 | 4.0 | c |  | 78.2 | 10.3 | a |  | 78.2 | 5.9 | a |  | **72.9** | **5.4** | b |  | 72.4 | 5.2 | b |
| Land use change (m^2^×10^-3^) | 41.0 | 5.3 | a |  | 43.8 | 5.2 | b |  | 38.0 | 3.1 | c |  | 39.0 | 2.1 | d |  | 41.6 | 5.0 | a |  | 45.1 | 4.2 | b |  | 38.6 | 2.7 | c |  | 40.3 | 3.5 | d |
| Water resources consumption (L) | 1697 | 515 | a |  | 1699 | 185 | a |  | **1337** | **190** | b |  | 1584 | 148 | c |  | 1943 | 535 | a |  | 2165 | 435 | b |  | **1510** | **197** | c |  | 1609 | 261 | d |
| Monetary cost (JPN) | 1015 | 159 | a |  | 1018 | 118 | a |  | 896 | 105 | b |  | 983 | 114 | c |  | 1161 | 190 | a |  | 1138 | 115 | a |  | 1040 | 120 | b |  | 1101 | 118 | c |

NRF, Nutrient Rich Food Index; JPN, Japanese yen.

Values changed by >10% from the observed value and statistically significant (P<0.05 by repeated-measures ANOVA with Bonferroni adjustment) are written in bold.

Values with different letters within a row (a,b,c,d) are significantly different from the values in observed diets or other optimised diets by repeated-measures ANOVA (two-sided P< 0·05 with Bonferroni adjustment). Values sharing the same letter are not significantly different.

*Those for whom a feasible solution could not be obtained were excluded from the analysis (39 younger and 31 older females).

†Optimised diets targeted to maximise the Nutrient Rich Food index 9.3.

‡Optimised diets targeted to minimise diet-related greenhouse gas emissions.

§Optimised diets target to achieve two goals (i.e., maximise the Nutrient Rich Food index 9.3 and minimise diet-related greenhouse gas emissions) with equal weight.

# Supplemental Table S12. Comparison of nutrient intake of observed diets and optimised diets among male participants with inefficient diets (per 2500 kcal)*

|  | Younger (aged 18-49 years; n=572) | | | | | | | | | | | | | | |
| --- | --- | --- | --- | --- | --- | --- | --- | --- | --- | --- | --- | --- | --- | --- | --- |
|  | Observed diets | |  |  | MaxNRF† | |  |  | MinGHGE‡ | |  |  | OPT2G§ | |  |
|  | Mean | SD |  |  | Mean | SD |  |  | Mean | SD |  |  | Mean | SD |  |
| Protein (g) | 86.0 | 11.9 | a |  | 89.6 | 7.0 | b |  | 81.2 | 6.8 | c |  | 83.3 | 7.9 | d |
| Total fat (g) | 81.8 | 14.2 | a |  | 73.5 | 9.1 | b |  | 60.5 | 9.2 | c |  | 60.6 | 9.1 | c |
| Saturated fat (g) | 23.8 | 5.2 | a |  | 20.3 | 3.7 | b |  | 16.9 | 3.4 | c |  | 16.2 | 3.5 | d |
| Carbohydrates (g) | 325 | 40 | a |  | 358 | 25 | b |  | 389 | 26 | c |  | 392 | 27 | c |
| Total fibre (g) | 13.1 | 2.7 | a |  | 17.6 | 3.5 | b |  | 16.5 | 3.7 | c |  | 19.0 | 3.8 | ab |
| Vitamin A (μgRAE) | 545 | 425 | a |  | 591 | 161 | b |  | 443 | 103 | c |  | 572 | 142 | d |
| Vitamin D (μg) | 7.2 | 3.6 | a |  | 8.9 | 3.1 | b |  | 7.1 | 3.3 | a |  | 8.2 | 4.1 | d |
| Vitamin E (mg) | 10.2 | 2.1 | a |  | 10.6 | 1.3 | b |  | 9.2 | 1.7 | c |  | 9.8 | 1.6 | d |
| Vitamin K (μg) | 235 | 95 | a |  | 295 | 71 | b |  | 269 | 97 | c |  | 328 | 89 | d |
| Thiamine (mg) | 1.3 | 0.3 | a |  | 1.3 | 0.1 | a |  | 1.1 | 0.1 | b |  | 1.1 | 0.1 | c |
| Riboflavin (mg) | 1.4 | 0.3 | a |  | 1.5 | 0.2 | b |  | 1.3 | 0.2 | c |  | 1.2 | 0.2 | c |
| Niacin (mg) | 21.4 | 5.1 | a |  | 22.0 | 2.6 | b |  | 18.2 | 2.3 | c |  | 20.2 | 2.3 | d |
| Vitamin B_6_ | 1.4 | 0.3 | a |  | 1.7 | 0.2 | b |  | 1.4 | 0.2 | a |  | 1.6 | 0.2 | c |
| Vitamin B_12_ (μg) | 6.0 | 3.4 | a |  | 7.1 | 2.4 | b |  | 9.3 | 4.2 | c |  | 9.3 | 5.1 | c |
| Folate (μg) | 335 | 96 | a |  | 401 | 71 | b |  | 316 | 61 | c |  | 316 | 61 | d |
| Pantothenic acid (mg) | 6.5 | 1.0 | a |  | 7.3 | 0.6 | b |  | 6.6 | 0.8 | a |  | 7.0 | 0.7 | d |
| Vitamin C (mg) | 95.9 | 38.2 | a |  | 117.7 | 24.6 | b |  | 83.2 | 25.4 | c |  | 106.1 | 25.3 | d |
| Sodium (g salt) | 12.1 | 2.2 | a |  | 11.2 | 1.3 | b |  | 11.6 | 1.5 | c |  | 10.6 | 1.2 | d |
| Potassium (mg) | 2616 | 481 | a |  | 3144 | 393 | b |  | 2565 | 421 | a |  | 2934 | 372 | d |
| Calcium (mg) | 498.4 | 137.0 | a |  | 679.3 | 109.7 | b |  | 520.4 | 133.2 | c |  | 663.2 | 151.4 | b |
| Magnesium (mg) | 278.1 | 47.4 | a |  | 349.9 | 50.3 | b |  | 307.5 | 62.5 | c |  | 357.0 | 55.8 | b |
| Iron (mg) | 8.4 | 1.5 | a |  | 10.3 | 1.7 | b |  | 9.7 | 2.7 | c |  | 10.8 | 2.4 | d |
| Zinc (mg) | 10.5 | 1.7 | a |  | 11.6 | 0.8 | b |  | 10.8 | 1.1 | c |  | 11.5 | 1.3 | b |
| Copper (mg) | 1.3 | 0.2 | a |  | 1.5 | 0.2 | b |  | 1.6 | 0.2 | c |  | 1.7 | 0.2 | d |
| Alcohol (g) | 10.8 | 19.7 | a |  | 2.6 | 4.5 | b |  | 4.5 | 7.5 | c |  | 3.1 | 4.6 | bc |
| Added sugar (g) | 35.7 | 22.6 | a |  | 28.7 | 11.7 | b |  | 18.5 | 8.1 | c |  | 20.7 | 8.7 | d |

Supplemental Table S12. *Continues*

|  | Older (aged 50-74 years; n=410) | | | | | | | | | | | | | |  |
| --- | --- | --- | --- | --- | --- | --- | --- | --- | --- | --- | --- | --- | --- | --- | --- |
|  | Observed diets | |  |  | MaxNRF† | |  |  | MinGHGE‡ | |  |  | OPT2G§ | |  |
|  | Mean | SD |  |  | Mean | SD |  |  | Mean | SD |  |  | Mean | SD |  |
| Protein (g) | 91.5 | 10.2 | a |  | 98.7 | 5.4 | b |  | 88.2 | 10.0 | c |  | 93.7 | 7.9 | d |
| Total fat (g) | 76.7 | 12.2 | a |  | 70.7 | 7.6 | b |  | 70.3 | 9.6 | b |  | 70.4 | 8.0 | b |
| Saturated fat (g) | 21.6 | 4.6 | a |  | 19.4 | 2.8 | b |  | 18.1 | 3.6 | c |  | 18.5 | 3.0 | c |
| Carbohydrates (g) | 313 | 42 | a |  | 339 | 28 | b |  | 306 | 48 | a |  | 342 | 26 | b |
| Total fibre (g) | 16.1 | 4.3 | a |  | 19.1 | 2.9 | b |  | 18.8 | 3.0 | b |  | 18.9 | 2.7 | b |
| Vitamin A (μgRAE) | 665 | 505 |  |  | 627 | 144 |  |  | 653 | 217 |  |  | 658 | 148 |  |
| Vitamin D (μg) | 10.5 | 4.7 | a |  | 14.7 | 3.5 | b |  | 11.0 | 2.7 | a |  | 12.6 | 3.6 | d |
| Vitamin E (mg) | 11.0 | 2.2 | a |  | 11.4 | 2.0 | b |  | 11.7 | 1.9 | b |  | 12.3 | 2.1 | d |
| Vitamin K (μg) | 294 | 123 | a |  | 380 | 60 | b |  | 387 | 78 | b |  | 360 | 71 | d |
| Thiamine (mg) | 1.3 | 0.2 | a |  | 1.3 | 0.1 | a |  | 1.1 | 0.2 | b |  | 1.3 | 0.2 | a |
| Riboflavin (mg) | 1.6 | 0.3 | ac |  | 1.7 | 0.2 | b |  | 1.5 | 0.2 | a |  | 1.6 | 0.2 | c |
| Niacin (mg) | 23.4 | 4.5 | a |  | 25.4 | 2.7 | b |  | 21.1 | 2.6 | c |  | 23.0 | 2.8 | a |
| Vitamin B_6_ | 1.7 | 0.3 | a |  | 2.0 | 0.2 | b |  | 1.6 | 0.3 | a |  | 1.8 | 0.3 | d |
| Vitamin B_12_ (μg) | 8.5 | 3.9 | a |  | 10.4 | 1.7 | b |  | 9.3 | 1.9 | c |  | 9.2 | 2.1 | c |
| Folate (μg) | 424 | 122 | a |  | 466 | 59 | b |  | 464 | 81 | b |  | 464 | 81 | d |
| Pantothenic acid (mg) | 7.1 | 1.1 | a |  | 8.1 | 0.6 | b |  | 7.3 | 0.8 | a |  | 7.6 | 0.8 | d |
| Vitamin C (mg) | 129.4 | 48.5 | a |  | 145.9 | 28.0 | b |  | 133.9 | 27.5 | a |  | 154.0 | 25.7 | d |
| Sodium (g salt) | 12.8 | 2.2 | a |  | 11.4 | 1.4 | b |  | 11.4 | 1.7 | b |  | 11.7 | 1.6 | d |
| Potassium (mg) | 3146 | 600 | a |  | 3622 | 378 | b |  | 3219 | 501 | a |  | 3516 | 409 | d |
| Calcium (mg) | 604.0 | 155.1 | a |  | 710.3 | 116.3 | b |  | 671.1 | 129.2 | c |  | 687.7 | 114.8 | bc |
| Magnesium (mg) | 335.4 | 56.5 | a |  | 396.1 | 35.5 | b |  | 357.1 | 47.2 | c |  | 375.0 | 36.5 | d |
| Iron (mg) | 9.8 | 1.8 | a |  | 11.5 | 1.0 | b |  | 10.6 | 1.6 | c |  | 11.3 | 1.1 | b |
| Zinc (mg) | 10.4 | 1.4 | a |  | 11.5 | 0.6 | b |  | 9.9 | 1.3 | c |  | 10.9 | 0.8 | d |
| Copper (mg) | 1.4 | 0.2 | a |  | 1.7 | 0.1 | b |  | 1.6 | 0.2 | c |  | 1.7 | 0.1 | b |
| Alcohol (g) | 22.8 | 24.2 | a |  | 11.8 | 11.8 | b |  | 37.9 | 36.2 | c |  | 13.5 | 15.1 | b |
| Added sugar (g) | 28.8 | 15.7 | a |  | 20.0 | 7.2 | b |  | 25.3 | 9.8 | c |  | 24.8 | 7.5 | c |

Values with different letters within a row (a,b,c,d) are significantly different from the values in observed diets or other optimised diets by repeated-measures ANOVA (two-sided P< 0·05 with Bonferroni adjustment). Values sharing the same letter are not significantly different.

For example in thiamine (younger males), there is no significant difference between observed diet and MaxNRF model (both with "a"), but MinGHGE (b) and OPT2G (c) are significantly different from each other and from observed diet and MaxNRF.

*Those for whom a feasible solution could not be obtained were excluded from the analysis (8 younger and 24 older male participants).

†Optimised diets targeted to maximise the Nutrient Rich Food index 9.3.

‡Optimised diets targeted to minimise diet-related greenhouse gas emissions.

§Optimised diets which target two goals (i.e., maximise the Nutrient Rich Food index 9.3. and minimise diet-related greenhouse gas emissions) with the same weight.

# Supplemental Table S13. Comparison of nutrient intake of observed diets and optimised diets among female participants with inefficient diets (per 2000 kcal)*

|  | Younger (aged 18-49 years; n=616) | | | | | | | | | | | | | |  |
| --- | --- | --- | --- | --- | --- | --- | --- | --- | --- | --- | --- | --- | --- | --- | --- |
|  | Observed diets | |  |  | MaxNRF† | |  |  | MinGHGE‡ | |  |  | OPT2G§ | |  |
|  | Mean | SD |  |  | Mean | SD |  |  | Mean | SD |  |  | Mean | SD |  |
| Protein (g) | 72.4 | 8.6 | a |  | 77.8 | 6.3 | b |  | 75.9 | 6.2 | c |  | 74.4 | 6.3 | d |
| Total fat (g) | 68.1 | 9.7 | a |  | 67.8 | 7.0 | a |  | 63.1 | 5.2 | b |  | 62.7 | 5.6 | b |
| Saturated fat (g) | 20.6 | 4.0 | a |  | 19.6 | 3.6 | b |  | 18.3 | 1.9 | c |  | 16.8 | 2.0 | d |
| Carbohydrates (g) | 259 | 27 | a |  | 264 | 19 | b |  | 277 | 13 | c |  | 279 | 15 | c |
| Total fibre (g) | 12.7 | 2.8 | a |  | 16.2 | 3.4 | b |  | 16.2 | 2.9 | b |  | 16.4 | 3.2 | b |
| Vitamin A (μgRTE) | 541 | 357 | a |  | 605 | 91 | b |  | 593 | 114 | b |  | 605 | 85 | b |
| Vitamin D (μg) | 6.9 | 3.6 | a |  | 8.4 | 2.9 | b |  | 10.2 | 2.5 | c |  | 8.0 | 3.1 | b |
| Vitamin E (mg) | 9.2 | 1.8 | a |  | 10.4 | 1.5 | b |  | 9.8 | 1.6 | c |  | 9.9 | 1.5 | c |
| Vitamin K (μg) | 227 | 92 | a |  | 280 | 77 | b |  | 382 | 105 | c |  | 278 | 78 | b |
| Thiamine (mg) | 1.05 | 0.19 | a |  | 1.04 | 0.12 | a |  | 1.00 | 0.15 | b |  | 0.97 | 0.12 | c |
| Riboflavin (mg) | 1.25 | 0.24 | a |  | 1.5 | 0.2 | b |  | 1.4 | 0.2 | c |  | 1.3 | 0.1 | d |
| Niacin (mg) | 17.4 | 3.7 | a |  | 18.1 | 2.8 | b |  | 17.2 | 3.5 | a |  | 17.0 | 3.0 | a |
| Vitamin B_6_ | 1.25 | 0.24 | a |  | 1.5 | 0.2 | b |  | 1.4 | 0.3 | c |  | 1.4 | 0.2 | d |
| Vitamin B_12_ (μg) | 5.27 | 3.08 | a |  | 6.7 | 2.0 | b |  | 6.3 | 2.3 | c |  | 6.5 | 2.1 | bc |
| Folate (μg) | 327 | 112 | a |  | 369 | 76 | b |  | 371 | 78 | c |  | 371 | 78 | d |
| Pantothenic acid (mg) | 5.73 | 0.85 | a |  | 6.8 | 0.7 | b |  | 6.7 | 0.7 | c |  | 6.4 | 0.6 | d |
| Vitamin C (mg) | 100 | 38 | a |  | 124 | 28 | b |  | 95 | 31 | c |  | 127 | 26 | b |
| Sodium (g salt) | 10.17 | 1.67 | a |  | 9.2 | 1.1 | b |  | 9.0 | 0.7 | c |  | 9.4 | 1.1 | d |
| Potassium (mg) | 2454 | 483 | a |  | 3046 | 423 | b |  | 2672 | 492 | c |  | 2914 | 416 | d |
| Calcium (mg) | 495 | 130 | a |  | 687 | 139 | b |  | 595 | 106 | c |  | 589 | 107 | c |
| Magnesium (mg) | 253 | 48 | a |  | 317 | 50 | b |  | 302 | 60 | c |  | 305 | 52 | c |
| Iron (mg) | 7.68 | 1.53 | a |  | 9.1 | 1.6 | b |  | 8.3 | 1.8 | c |  | 8.8 | 1.6 | b |
| Zinc (mg) | 8.57 | 1.12 | a |  | 9.2 | 0.9 | b |  | 8.6 | 0.5 | b |  | 8.6 | 0.7 | b |
| Copper (mg) | 1.09 | 0.17 | a |  | 1.3 | 0.2 | b |  | 1.3 | 0.2 | c |  | 1.3 | 0.2 | bc |
| Alcohol (g) | 5.14 | 12.44 | a |  | 0.8 | 1.6 | b |  | 0.5 | 0.8 | b |  | 0.6 | 0.6 | b |
| Added sugar (g) | 33.0 | 15.0 | a |  | 25.2 | 8.0 | b |  | 23.2 | 5.1 | c |  | 22.5 | 8.4 | d |

Supplemental Table S13. *Continues*

|  | Older (aged 50-74 years; n=440) | | | | | | | | | | | | | |  |
| --- | --- | --- | --- | --- | --- | --- | --- | --- | --- | --- | --- | --- | --- | --- | --- |
|  | Observed diets | |  |  | MaxNRF† | |  |  | MinGHGE‡ | |  |  | OPT2G§ | |  |
| (per 2000 kcal) | Mean | SD |  |  | Mean | SD |  |  | Mean | SD |  |  | Mean | SD |  |
| Protein (g) | 76.8 | 8.6 | a |  | 77.1 | 7.1 | a |  | 78.6 | 5.5 | b |  | 74.8 | 6.6 | c |
| Total fat (g) | 67.2 | 9.3 | a |  | 64.3 | 5.3 | b |  | 64.1 | 4.5 | b |  | 61.2 | 5.8 | c |
| Saturated fat (g) | 19.6 | 3.7 | a |  | 17.9 | 2.0 | b |  | 18.7 | 1.8 | c |  | 17.5 | 2.1 | b |
| Carbohydrates (g) | 258 | 28 | a |  | 276 | 16 | b |  | 271 | 12 | c |  | 285 | 17 | d |
| Total fibre (g) | 15.3 | 3.3 | a |  | 17.7 | 2.6 | b |  | 18.1 | 2.9 | bc |  | 18.4 | 3.5 | c |
| Vitamin A (μgRTE) | 660 | 405 | a |  | 703 | 160 | a |  | 770 | 226 | b |  | 838 | 237 | c |
| Vitamin D (μg) | 9.1 | 4.4 | a |  | 8.5 | 2.7 | a |  | 9.0 | 2.7 | a |  | 7.6 | 2.1 | b |
| Vitamin E (mg) | 10.2 | 1.9 | a |  | 11.4 | 1.0 | b |  | 10.3 | 2.0 | a |  | 10.8 | 1.2 | c |
| Vitamin K (μg) | 275 | 108 | a |  | 294 | 74 | b |  | 394 | 79 | c |  | 306 | 89 | b |
| Thiamine (mg) | 1.1 | 0.2 | ac |  | 1.2 | 0.1 | b |  | 1.1 | 0.1 | a |  | 1.1 | 0.1 | c |
| Riboflavin (mg) | 1.4 | 0.2 | a |  | 1.4 | 0.2 | b |  | 1.5 | 0.1 | b |  | 1.4 | 0.2 | a |
| Niacin (mg) | 19.1 | 3.6 | a |  | 20.4 | 2.9 | b |  | 17.4 | 2.5 | c |  | 18.0 | 2.3 | d |
| Vitamin B_6_ | 1.4 | 0.3 | a |  | 1.5 | 0.2 | b |  | 1.5 | 0.2 | b |  | 1.5 | 0.2 | b |
| Vitamin B_12_ (μg) | 6.9 | 3.3 | a |  | 6.2 | 1.7 | bc |  | 6.4 | 1.9 | b |  | 5.9 | 1.8 | c |
| Folate (μg) | 406 | 104 | a |  | 429 | 62 | b |  | 422 | 70 | bcd | | 422 | 70 | ad |
| Pantothenic acid (mg) | 6.2 | 0.9 | a |  | 6.6 | 0.7 | b |  | 7.3 | 0.6 | c |  | 6.7 | 0.6 | d |
| Vitamin C (mg) | 137 | 45 | a |  | 170 | 24 | b |  | 139 | 35 | a |  | 160 | 26 | c |
| Sodium (g salt) | 10.9 | 1.8 | a |  | 10.2 | 1.2 | b |  | 9.7 | 1.0 | c |  | 9.4 | 1.0 | d |
| Potassium (mg) | 2939 | 527 | a |  | 3298 | 311 | b |  | 3248 | 391 | bc |  | 3216 | 400 | c |
| Calcium (mg) | 585 | 130 | a |  | 683 | 96 | b |  | 695 | 91 | b |  | 648 | 110 | c |
| Magnesium (mg) | 297 | 47 | a |  | 369 | 39 | b |  | 325 | 43 | c |  | 331 | 42 | c |
| Iron (mg) | 8.9 | 1.5 | a |  | 9.6 | 0.9 | b |  | 9.1 | 1.1 | c |  | 9.1 | 1.1 | ac |
| Zinc (mg) | 8.8 | 1.1 | a |  | 9.1 | 0.9 | b |  | 9.1 | 0.5 | b |  | 8.7 | 0.5 | a |
| Copper (mg) | 1.2 | 0.2 | a |  | 1.4 | 0.1 | b |  | 1.4 | 0.1 | b |  | 1.4 | 0.1 | b |
| Alcohol (g) | 5.4 | 11.0 | a |  | 0.8 | 1.0 | b |  | 2.0 | 3.6 | c |  | 0.7 | 1.0 | b |
| Added sugar (g) | 30.0 | 12.3 | a |  | 30.2 | 8.5 | a |  | 22.8 | 6.7 | b |  | 30.0 | 9.4 | a |

Values with different letters within a row (a,b,c,d) are significantly different from the values in observed diets or other optimised diets by repeated-measures ANOVA (two-sided P< 0·05 with Bonferroni adjustment). Values sharing the same letter are not significantly different.

For example, in protein (older females), there is no significant difference between observed diet and MaxNRF model (both with "a"), but MinGHGE (b) and OPT2G (c) are significantly different from each other and from observed diet and MaxNRF.

*Those for whom a feasible solution could not be obtained were excluded from the analysis (39 younger and 31 older female participants) .

†Optimised diets targeted to maximise the Nutrient Rich Food index 9.3.

‡Optimised diets targeted to minimise diet-related greenhouse gas emissions.

§Optimised diets which target two goals (i.e., maximise the Nutrient Rich Food index 9.3. and minimise diet-related greenhouse gas emissions) with the same weight.

# Figure S1. Mean change (g) in food intake from observed to optimised diets among participants with inefficient diets

MaxNRF, optimised diets targeted to maximise the Nutrient Rich Food index 9.3; MinGHGE, optimised diets targeted to minimise diet-related greenhouse gas emissions; OBS, observed diets; OPT2G, optimised diets which target two goals (i.e., maximise the Nutrient Rich Food index 9.3. and minimise diet-related greenhouse gas emissions) with the same weight.

The difference in food intake (g) was calculated for each individual as $\left( intake in \mathrm{the}optimised diet-intake in \mathrm{the}observed diet \right)$, and the mean difference was then calculated across individuals within sex-age group. For visualisation purposes, cells are colour-shaded such that positive values are green and negative values are red. Greater colour intensity reflects larger absolute changes. Participants for whom a feasible solution could not be obtained were excluded from the analysis (8 younger males, 24 older males, 39 younger females, and 31 older females).
