# Supplementary material for: Optimizing Japanese dietary patterns to improve nutritional quality, reduce environmental impact under affordability constraints in younger and older adults from the nationwide MINNADE study
Source: Eur J Nutr. 2026 Oct 13;65(8):282. doi: 10.1007/s00394-026-04129-x (PMC13661754; doi:10.1007/s00394-026-04129-x)
Supplement: Supplementary file 2 [file 394_2026_4129_MOESM2_ESM.docx]

# Appendix A, Description of basic Data Envelopment Analysis models

Input-oriented and output-oriented Banker, Charnes and Cooper (BCC) models^(1)^ for Data Envelopment Analysis (DEA) was used. Following the description of the DEA model was according to the previous study by Kanellopoulos, et al ^(2)^.

Input-oriented DEA:

| $min\left\{ \theta-\varepsilon\left( \sum_{\iota} s_{i}^{in}+\sum_{j} s_{j}^{ot} \right) \right\}$ | $\theta\in\left\{ 0\ldots1 \right\}$ |
| --- | --- |
| *s.t.:* |  |
| $\sum_{k} y_{jk}\lambda_{k}-y_{j}^{0}-s_{j}^{ot}=0$ | $\forall j$ |
| $\sum_{k} x_{ik}\lambda_{k}-\theta x_{i}^{0}+s_{i}^{in}=0$ | $\forall i$ |
| $\sum_{k} \lambda_{k}=1$ |  |
| $\lambda_{k}, s_{j}^{ot}, s_{i}^{in}\geq0$ | $\forall k,j, i$ |

Output-oriented DEA:

| $max\left\{ \varphi+\varepsilon\left( \sum_{\iota} s_{i}^{in}+\sum_{j} s_{j}^{ot} \right) \right\}$ |  |
| --- | --- |
| *s.t.:* |  |
| $\sum_{k} y_{jk}\lambda_{k}-{\varphi y}_{j}^{0}-s_{j}^{ot}=0$ | $\forall j$ |
| $\sum_{k} x_{ik}\lambda_{k}-x_{i}^{0}+s_{i}^{in}=0$ | $\forall i$ |
| $\sum_{k} \lambda_{k}=1$ |  |
| $\lambda_{k}, s_{j}^{ot}, s_{i}^{in}\geq0$ | $\forall k,j, i$ |

Where $\theta$ is the efficiency score of the evaluated diet calculated with the input-oriented DEA model (DEA-efficient diets get the value of 1), *λ_k_* is a decision variable and the weight of diet *k* in the alternative of the evaluated diet, *s_j_^ot^* is the slack decision variable capturing the deviation between the intake of ‘dietary components to increase’ food or nutrient *j* of the alternative diet and that of the current diet, *s_i_^in^* is the slack decision variable that captures the deviation between the intake of ‘dietary components to decrease’ nutrient *i* of the current diet and that of the alternative diet, *ε* is a marginal (i.e. very small) positive number, *x_ik_* is intake of ‘dietary components to decrease’ food or nutrient *i* in diet *k*, *y_jk_* is intake of ‘dietary components to increase’ food or nutrient *j* in diet *k*, *x_i_^0^* is intake of ‘dietary components to decrease’ nutrient *i* in the evaluated diet, and *y_j_^0^* is intake of ‘dietary components to increase’ for or nutrient *j* in the evaluated diet.

*φ* is the efficiency score of the evaluated diet calculated with the output-oriented DEA model. The higher the value of *φ* the higher the efficiency of the evaluated diet. To normalize the efficiency scores of the output-oriented DEA to values from 0 (i.e. lowest efficiency) to 1 (i.e. highest efficiency), efficiency scores were reported as *φ^-1^*. Both the input-oriented DEA and the output-oriented DEA models were solved in two stages following Cooper et al. ^(1)^

In the benchmarking process of diets, the efficiency scores $\theta$ (0≤$\theta$≤1) and *φ^-1^* (0≤*φ^-1^* ≤1) were calculated^(2)^, respectively, for each individual following the method by Cooper, et al ^(1)^. As a result of the analysis, the diet with $\theta$ = 1 or *φ^-1^* = 1 was identified as ‘DEA-efficient diets.’ It was confirmed that the same participants were identified as with ‘DEA-efficient diets’ in both input- and output-oriented DEA models.

1. Cooper WW, Seiford LM & Tone K (2007) *Data Envelopment Analysis*. New York: Springer.

2. Kanellopoulos A, Gerdessen JC, Ivancic A, et al. (2020) Designing healthier and acceptable diets using data envelopment analysis. *Public Health Nutr.* **23**, 2290–2302.

# Appendix B, Sets, indices, model parameters, and decision variables for Appendix C

## Table B1. Sets and indices

| Index | Description |
| --- | --- |
| *l* | Index for different objectives |
| *k* | Index of modelled diets of individuals |
| *j* | Food groups and nutrients included in the benchmarking with DEA as dietary components to increase |
| *i* | Food groups and nutrients included in the benchmarking with DEA as dietary components to decrease |
| *f* | Food groups for calculating deviation between food consumption in modelled diets and the observed diets |

## Table B2: Model parameters

| Parameter | Description | Units |
| --- | --- | --- |
| GHGE_k_ | Diet-related greenhouse gas emissions of diet *k* | gram CO_2_-eq |
| NRF_k_ | Nutrient Rich Food index 9.3 score of diet *k* | no unite |
| COST_k_ | Monetary diet cost of diet _k_ | Japanese Yen |
| VitA_k_ | Vitamin A intake for diet _k_ | μgRAE |
| VitA_95_ | Sex-specific 95th percentile of observed vitamin A intake | μgRAE |
| $V_{l}^{max}$ | The maximum observed value for objective *l* | no unit for $V_{NRF}^{max}$, g CO_2_-eq for $V_{GHGE}^{max}$ |
| $V_{l}^{min}$ | The minimum observed value for objective *l* | no unit for $V_{NRF}^{min}$, g CO_2_-eq for $V_{GHGE}^{min}$ |
| $w_{l}$ | The importance weight of objective function *l* | - |
| $y_{jk}$ | The amount of intake of dietary components to increase *j* in the diet *k* | gram |
| $y_{j}^{0}$ | The amount of intake of dietary components to increase *j* in the diet of the evaluated diet | gram |
| $x_{ik}$ | The amount of intake of dietary components to decrease *i* in the diet *k* | gram |
| $x_{i}^{0}$ | The amount of intake of dietary components to decrease *i* in the evaluated diet | gram |

## Table B3: Decision variables

| Variable | Description | Units |
| --- | --- | --- |
| λ_k_ | The proportion of diet k in the modelled diet | - |
| *V_l_* | Value of objective l, namely, V_acceptability_ the total deviation of food item intakes between modelled and observed diet, V_NRF_ the NRF score of the modelled diet, V_cost_ the total cost of the modelled diet and V_GHGE_ the total greenhouse gas emissions of the modelled diet | gram for V_accecibility_, no unit for V_NRF_, Japanese yen for V_cost_, g CO_2_-eq for V_GHGE_ |

# Appendix C: Formulas

| $minimise \left\{ w_{NRF}\times\left( \frac{V_{NRF}}{V_{NRF}^{max}-V_{NRF}^{min}} \right)+w_{GHGE}\times\left( \frac{V_{GHGE}}{V_{GHGE}^{max}-V_{GHGE}^{min}} \right) \right\}$ | (1) |
| --- | --- |

Subject to:

| $V_{NRF}=\sum_{k} {NRF}_{k}\lambda_{k}$ |  | (2) | |
| --- | --- | --- | --- |
| $V_{GHGE}=\sum_{k} {GHGE}_{k}\lambda_{k}$ |  | (3) | |
| $\sum_{k} \lambda_{k}y_{jk}-y_{j}^{0}\geq0$ | $\forall j$ | (4) |  |
| $\sum_{k} \lambda_{k}x_{ik}-x_{i}^{0}\leq0$ | $\forall i$ | (5) |  |
| $\sum_{k} \lambda_{k}= 1$ |  | (6) |  |
| $\lambda_{k}\geq0$ | $\forall k$ | (7) |  |
| $\sum_{k} {VitA}_{k}\lambda_{k}\leq$VitA_95_ |  | (8) |  |
| $\sum_{k} {COST}_{k}\lambda_{k}\leq{COST}_{k}\times1.05$ |  | (9) |  |

Value of $w_{NRF}$ and $w_{GHGE}$:

MaxNRF model: $w_{NRF}=0$ and $w_{GHGE}=1$

MinGHGE model: $w_{NRF}=1$ and $w_{GHGE}=0$

OPT2G model: $w_{NRF}=1$ and $w_{GHGE}=1$

(4) and (5) are included as constraints of Intake directionality.

(6) was included as an energy balance constraint.

(8) was included as a constraint of vitamin A.

(9) was included as a cost constraint.
